# Rejuvenation of mesenchymal stem cells by extracellular vesicles inhibits the elevation of reactive oxygen species

**DOI:** 10.1038/s41598-020-74444-8

**Published:** 2020-10-14

**Authors:** Vuong Cat Khanh, Toshiharu Yamashita, Kinuko Ohneda, Chiho Tokunaga, Hideyuki Kato, Motoo Osaka, Yuji Hiramatsu, Osamu Ohneda

**Affiliations:** 1 Laboratory of Regenerative Medicine and Stem Cell Biology, Graduate School of Comprehensive Human Science, University of Tsukuba, Tsukuba, 305-8575 Japan; 2 Department of Medical Biochemistry, Graduate School of Medicine, Tohoku University, Miyagi, Japan; 3 Department of Cardiovascular Surgery, University of Tsukuba, Tsukuba, Japan

**Keywords:** Cell biology, Molecular biology, Stem cells

## Abstract

Aging induces numerous cellular disorders, such as the elevation of reactive oxygen species (ROS), in a number type of cells, including mesenchymal stem cells (MSCs). However, the correlation of ROS and impaired healing abilities as well as whether or not the inhibition of elevating ROS results in the rejuvenation of elderly MSCs is unclear. The rejuvenation of aged MSCs has thus recently received attention in the field of regenerative medicine. Specifically, extracellular vesicles (EVs) act as a novel tool for stem cell rejuvenation due to their gene transfer ability with systemic effects and safety. In the present study, we examined the roles of aging-associated ROS in the function and rejuvenation of elderly MSCs by infant EVs. The data clearly showed that elderly MSCs exhibited the downregulation of superoxide dismutase (SOD)1 and SOD3, which resulted in the elevation of ROS and downregulation of the MEK/ERK pathways, which are involved in the impairment of the MSCs’ ability to decrease necrotic area in the skin flap model. Furthermore, treatment with the antioxidant Edaravone or co-overexpression of SOD1 and SOD3 rescued elderly MSCs from the elevation of ROS and cellular senescence, thereby improving their functions. Of note, infant MSC-derived EVs rejuvenated elderly MSCs by inhibiting ROS production and the acceleration of cellular senescence and promoting the proliferation and in vivo functions in both type 1 and type 2 diabetic mice.

## Introduction

Tissue stem cells, which possess stemness and the ability to differentiate into multiple cell types, are an attractive source for stem cell therapy due to their safety and lack of ethical issues^1^. Given their lifelong persistence inside differentiated tissues, tissue stem cells are affected by common physiological events that occur in those tissues, such as aging^2^. The aging of tissue stem cells is recognized when stem cells lose their functions due to reductions in their self-renewal and proliferation abilities, responsiveness to tissue injury, and differentiation abilities along with the induction of cellular senescence and apoptosis^2–5^. Substantial evidence of the molecular processes that control the age-dependent progressive deterioration of stem cell functions has been reported, including the accumulation of toxic metabolites such as reactive oxygen species (ROS), mitochondrial dysfunction, DNA damage, protein homeostasis, extracellular signaling, epigenetic remodeling, and cellular depletion and senescence^2^.

ROS, including free radical and non-free radical oxygen-containing molecules, are generated as the natural by-products of normal cellular metabolism or from cellular responsiveness to extrinsic paracrine and endocrine mediators^6^. In stem cells, ROS regulate the responsiveness of stem cells to the metabolic and environmental signals, keeping stem cells in a quiescent state or allowing them to continue cycling^7^. The excess of ROS is associated with aging due to the disrupted regulation of cellular metabolisms results in cellular damage or malfunction has been suggested^8,9^. However, the details regarding the correlation of ROS and the aging-related decrease in the function of stem cells are unclear^10^. Because ROS is involved in numerous types of aging-associated diseases, clarifying the underlying mechanisms related to the elevation of ROS and the anti-oxidative defense is extremely important for understanding the intrinsic stem cell aging process^11^.

Over the past decade, the concept of the rejuvenation of stem cells has encouraged many studies to reset aged stem cells to a younger state^12^. With the development of cell reprogramming techniques, the generation of pluripotent stem cells from tissue stem cells has been considered the ideal strategy^13^. However, the application of reprogramming techniques to human subjects has raised ethical issues and prompted safety concerns, as the pluripotent state carries a risk of inducing cancer^13^. As such, much effort has been made to propose safer strategies involving interference in the aging-associated molecular alterations of tissue stem cells, such as via pharmaceutical administration and genetic modification^14^.

The main principle of pharmacological manipulation is targeting the specific pathways involved in the aging process, such as the mTOR/PI3K, Wnt/β-catenin, and STAT3/NFκB pathways^15^. Genetic intervention includes reversing DNA damage, telomerase reactivation, epigenetic regulation (e.g. repair of DNA methylation and histone modification), all conventional approaches that have proven effective for improving the function of several types of stem cells, such as muscle, cardiac, and hematopoietic stem cells^16^. However, both pharmacological and genetic manipulation have limited efficacy, as they can only improve a few specific functions, thus requiring the manipulation of a combination of genes or pathways for a comprehensive approach to rejuvenating stem cells^15,16^.

Recent advances in genetic modification have focused on the application of extracellular vesicles (EVs), as these are less invasive than conventional methods using a viral vector, have improved safety, and can target a combination of genes or pathways of interest^17^. EVs, including exosomes and microvesicles, are nano-sized heterogeneous membrane-vesicles of endosomal and plasma membrane origin and contain a number of biological molecules, such as DNA, small RNAs, proteins, and lipids^18,19^. The role of EVs as a tool for genetic transfer was suggested by a previous report showing that mRNA/miRNA contained inside exosomes from mouse mast cells could be delivered to recipient cells and be functional in the new location^20^. Notably, EVs derived from induced pluripotent stem cells that were applied to the culture of senescent MSCs reduced the cells’ elevated ROS and alleviated the cellular senescence of aging MSCs^21^, which supports the notion of rejuvenating aging cells using EVs. However, whether or not EVs derived from young subjects are able to rejuvenate aged tissue-derived stem cells—as well as the mechanisms involved—remains unclear.

In the present study, we examined the correlation between aging and the expression of ROS in human adipose tissue-derived (AT-) MSCs and the cellular functions by comparing AT-MSCs derived from elderly subjects with those from the infants. In addition, we also examined the roles of infant AT-MSC-derived EVs and the ROS-related mechanisms involved in the rejuvenation of elderly AT-MSCs.

## Results

### The aging-associated ROS and impaired functions of elderly donor-derived AT-MSCs to decrease the necrotic area of the flap mouse model

**Figure 1:**
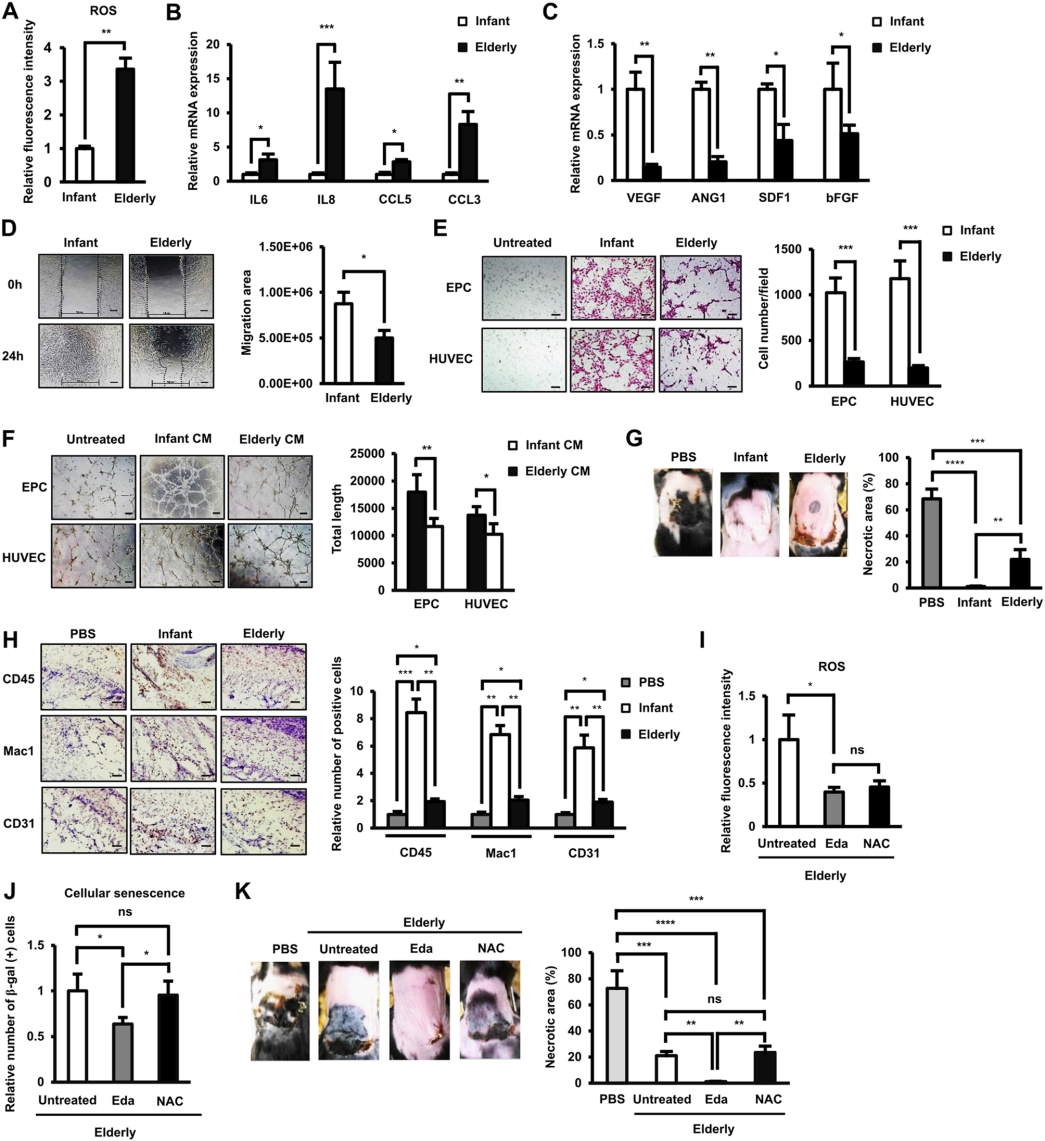
The aging-associated ROS and impaired functions of elderly donor-derived AT-MSCs to decrease the necrotic area of the flap mouse model. (**A**) The ROS expression in infant and elderly AT-MSCs. (n = 5). (**B**) The mRNA expression of pro-inflammatory factors in infant and elderly AT-MSCs (n = 5). (**C**) The mRNA expression of wound healing-related growth factors in infant and elderly AT-MSCs (n = 5). (**D**) The migration scratch assay of infant and elderly AT-MSCs, magnification 4 ×. Bar indicates 200 μM (n = 5). (**E**) The transwell assay of EPCs and ECs co-cultured with infant and elderly AT-MSCs magnification 10 ×. Bar indicates 200 μM (n = 5). (**F**) The tube formation of EPCs and ECs in the infant and elderly AT-MSC-conditioned medium, magnification 4 ×. Bar indicates 200 μM (n = 5). (**G**) Transplantation of infant and elderly AT-MSCs to an in vivo streptozotocin-induced diabetic ischemic flap mouse model (n = 5). (**H**) Immunohistochemical staining with PE-labeled anti-CD45 and Mac 1 on the third day and CD-31 on the seventh day of transplantation of the necrotic areas. The brown dots indicate positive cells, magnification 10 ×. Bar indicates 200 μM (n = 5). (**I**) The expression of ROS in elderly AT-MSCs in the presence of antioxidants (n = 5). (**J**) The cellular senescence of elderly AT-MSCs in the presence of antioxidants (n = 5). (**K**) Transplantation of elderly AT-MSCs and antioxidant-treated elderly AT-MSCs to an in vivo diabetic ischemic flap mouse model. Edaravone (Eda, 20 µM) and N-acetylcystein (NAC 2 mM) were used as antioxidants with a treatment time of 24 h (n = 5). In all above experiments, infant AT-MSCs and elderly AT-MSCs were derived from 5 different donors, respectively (n = 5). AT-MSCs were at passage 3 to passage 8, and the comparison was conducted with infant AT-MSCs and elderly AT-MSCs at the same passage number. The data represent the mean ± SD, ***P < 0.001, **P < 0.01, *P < 0.05, *ns* no significance. The experiments were performed in triplicate.

Firstly, AT-MSCs from infant and elderly donors were isolated and characterized. As the results, both infant and elderly AT-MSCs showed the typical characteristics of MSCs such as: fibroblast-like morphology, expressed the MSC marker profile, and possessed the abilities to proliferate and differentiate to adipocytes, osteocytes, and chondrocytes (Supplementary Figure 1A-D). The elevation of ROS is thought to be correlated with aging^11^; however, the role of ROS in the cellular functions of aging AT-MSCs has not yet been clarified. To investigate this issue, we first compared the expression of ROS from infant- and elderly-derived AT-MSCs. The results showed a significantly higher ROS level in elderly-derived AT-MSCs (3.36-fold higher) than in the infant groups (Fig. 1A). In addition, the expression of growth factors in AT-MSCs was altered due to aging. Indeed, the data showed the upregulation of proinflammatory cytokines (IL6, IL8) and chemokines (CCL5, CCL3) (Fig. 1B) in elderly AT-MSCs compared to those in the infant group. In contrast, other homeostatic factors, such as angiogenic and homing factors, showed an impaired expression in elderly AT-MSCs (Fig. 1C).

We next analyzed the migration ability of different age group-derived AT-MSCs by an in vitro scratch assay. The results showed that elderly AT-MSCs exhibited an impaired mobility compared to the infant group. After 24-h incubation, elderly AT-MSCs covered less than twice the area of infant AT-MSCs (Fig. 1D), indicating a decreased migratory ability in proportion to age. Because the impaired expression of growth factors responsible for homing (SDF1) and angiogenesis (VEGF, Ang1, bFGF) was observed in elderly AT-MSCs (Fig. 1C), which are involved in the regulation of EC and EPC functions, we next examined the different in vitro paracrine effects of infant and elderly AT-MSCs on EPCs and ECs. While EPCs were isolated from the umbilical cord blood and the characteristics were shown in Supplementary Figure 1E–H, HUVEC was used as the ECs. First, we analyzed the effects of AT-MSCs on recruiting EPCs and ECs under a transwell-coculture condition. Consistent with the low expression of SDF1, elderly AT-MSCs showed less ability to recruit EPCs and ECs, which are cells with a high expression of CXCR4 as a receptor of SDF1 ligand^22^, than infant AT-MSCs (EPCs: 3.8-fold decrease, ECs: 5.9-fold decrease, Fig. 1E). In addition, the ability of infant and elderly AT-MSCs to support the angiogenic functions of EPCs and ECs was compared by the tube formation assay using MSC-conditioned medium (CM). As expected, EPCs and ECs incubated in elderly CM showed a lower ability to form tubes than those incubated in infant CM (Fig. 1F).

Next, to address how aging affects the functions of AT-MSCs in vivo, we conducted transplantation experiments using an ischemic flap mouse model with streptozocin-induced diabetes, which have impaired wound healing. The data showed the higher necrotic area remaining in mice treated with elderly AT-MSCs than in those treated with the infant group; after 7 days of injection, the transplantation of infant AT-MSCs significantly decreased the necrotic area in flap mouse model while the transplantation of elderly AT-MSCs showed the impaired functions (necrotic area in infant AT-MSC-transplanted mice: 1.01%, necrotic area in elderly AT-MSC-transplanted mice: 21.9%, Fig. 1G). In addition, greater numbers of CD45- and Mac1-positive cells on day 3 and CD31-positive cells on day 7 post-transplantation were observed in the subcutaneous region of infant AT-MSC-injected mice than in the same region of PBS- and elderly AT-MSC-injected mice (CD45: 4.4-fold higher, Mac1: 3.4-fold higher, CD31: 3.1-fold higher in mice transplanted with infant AT-MSCs compared to those transplanted with elderly AT-MSCs, Fig. 1H). This indicates the impaired recruitment of inflammatory cells and neovascularization in mice transplanted with elderly AT-MSCs.

In order to examine the direct relationship of elevated ROS levels with the healing functions of AT-MSCs, we treated elderly AT-MSCs with the antioxidants edaravone and NAC and examined their functions on the flap mouse model. The antioxidants were confirmed to reduce the ROS expression in elderly AT-MSCs (Fig. 1I). Of note, treatment with Edaravone reduced the number of β-galactosidase (gal)-positive elderly AT-MSCs which related to cellular senescence, while NAC showed no such effects (Fig. 1J). Next, we observed the effects of antioxidant treatment on the ability of elderly AT-MSCs to decrease the necrotic area in type 1 diabetes mellitus (T1DM) mice. NAC showed no marked effects on the ability of elderly AT-MSCs to decrease necrotic area, while Edaravone-treated elderly AT-MSCs showed a significantly enhanced ability to decrease necrotic area, compared to control elderly AT-MSCs (necrotic area of mice with elderly AT-MSCs: 21.12%, necrotic area of mice with Edaravone-treated elderly AT-MSCs: 1.11%, necrotic area of mice with NAC-treated elderly AT-MSCs: 23.54%, Fig. 1K).

Taken together, these data showed that aging increased the accumulation of ROS and altered the gene profile of AT-MSCs, which impaired their migratory ability and paracrine effects on EPCs and ECs. Of note, the elevation of ROS was shown to be directly involved in the declined functions of AT-MSCs in vivo.

### The role of superoxide dismutase (SOD)1 and SOD3 antioxidant enzymes in the functions of AT-MSCs

**Figure 2:**
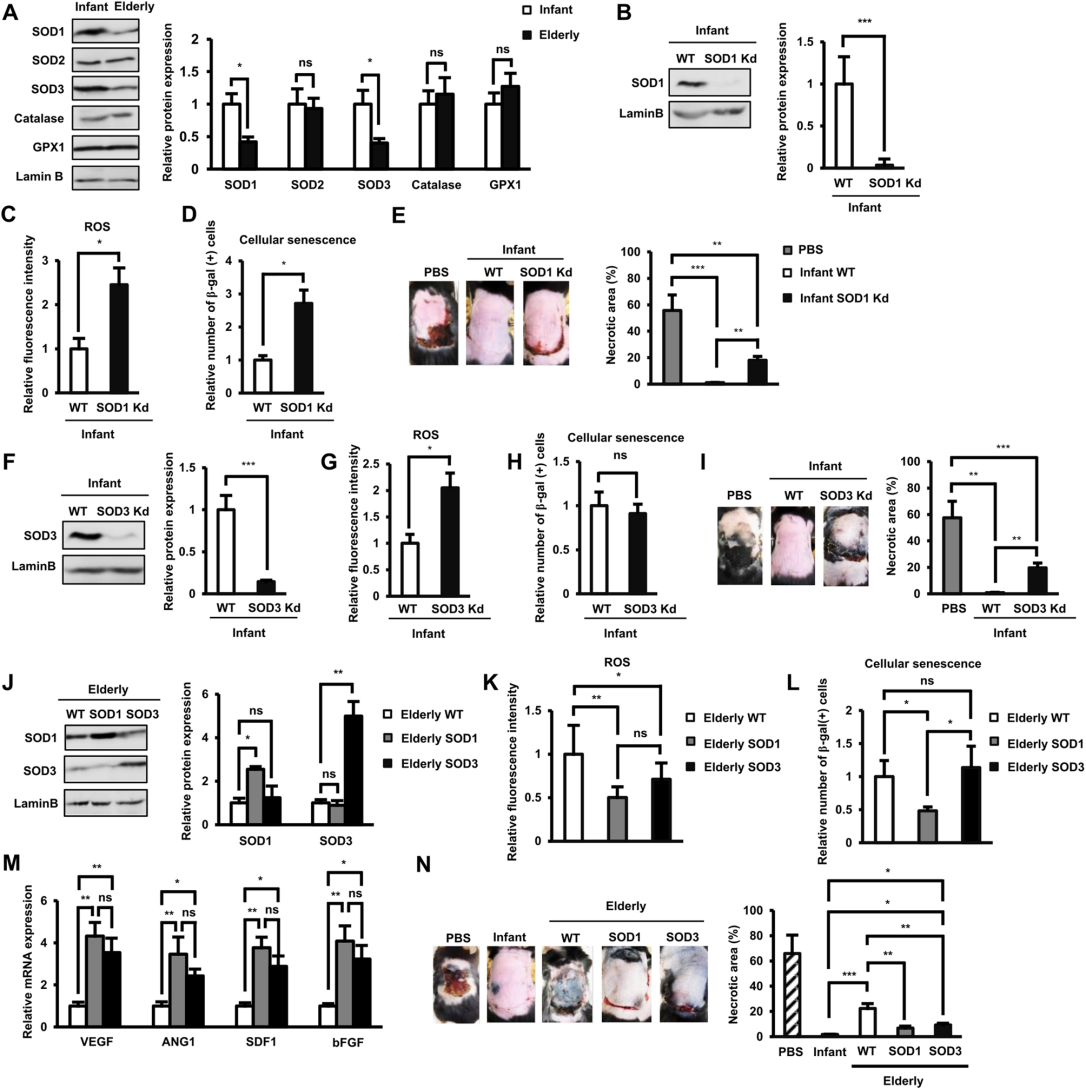
The role of SOD1 and SOD3 antioxidant enzymes in the functions of AT-MSCs. (**A**) The protein expression of antioxidant enzymes in infant and elderly AT-MSCs. Infant AT-MSCs and elderly AT-MSCs were derived from 5 different donors, respectively (n = 5) and the comparison was conducted with infant AT-MSCs and elderly AT-MSCs at the same passage number. (**B**) The protein expression of wild-type and SOD1 knockdown infant AT-MSCs. The knockdown experiments were conducted with infant AT-MSCs from 3 different donors (n = 3). (**C**) The expression of ROS in wild-type and SOD1 knockdown infant AT-MSCs (n = 3). (**D**) The cellular senescence of wild-type and SOD1 knockdown infant AT-MSCs (n = 3). (**E**) Transplantation of wild-type and SOD1 knockdown infant AT-MSCs to an in vivo streptozotocin-induced diabetic ischemic flap mouse model (n = 3). (**F**) The protein expression of wild-type and SOD3 knockdown infant AT-MSCs. (**G**) The expression of ROS in wild-type and SOD3 knockdown infant AT-MSCs (n = 3). (**H**) The cellular senescence of wild-type and SOD3 knockdown infant AT-MSCs (n = 3). (**I**) Transplantation of wild-type and SOD3 knockdown infant AT-MSCs to an in vivo streptozotocin-induced diabetic ischemic flap mouse model (n = 3). (**J**) The protein expression of wild-type elderly AT-MSCs or with the overexpression of SOD1 or SOD3. The overexpression experiments were conducted with elderly AT-MSCs from 3 different donors (n = 3). (**K**) The ROS expression in wild-type elderly AT-MSCs or with the overexpression of SOD1 or SOD3 (n = 3). (**L**) The cellular senescence of wild-type elderly AT-MSCs or with the overexpression of SOD1 or SOD3 (n = 3). (**M**) The mRNA expression of wound healing-related growth factors in wild-type elderly AT-MSCs or with the overexpression of SOD1 or SOD3 (n = 3). (**N**) Transplantation of wild-type elderly AT-MSCs or with the overexpression of SOD1 or SOD3 to a streptozotocin-induced in vivo diabetic ischemic flap mouse model (n = 3). WT: wild-type, SOD1 Kd: knockdown of SOD1, SOD3 Kd: knockdown of SOD3, SOD1: overexpression of SOD1, SOD3: overexpression of SOD3. The data represent the mean ± SD. ***P <0.001, **P < 0.01, *P < 0.05, *ns* no significance. The experiments were performed in triplicate. Full-length Western blots are shown in Supplementary Figure S3.

In order to clarify the mechanisms related to the ROS elevation in elderly AT-MSCs, we assessed the expression of several enzymes involved in antioxidant defense. In contrast to the increase in ROS, at protein levels, the expression of antioxidant enzymes, including SOD1 and SOD3, decreased with aging (SOD1: 2.3-fold decrease in protein level, and SOD3: 2.5-fold decrease in protein level, n = 4, *p* < 0.05, Fig. 2A).

To clarify whether SOD1 or SOD3 regulates the aging-associated ROS, cellular senescence, and in vivo functions of AT-MSCs, we conducted the knockdown of SOD1 or SOD3 in infant AT-MSCs and examined the subsequent effects on these cells. The knockdown of either SOD1 or SOD3 maintained the proliferative ability and expression of MSC markers of infant AT-MSCs (Supplementary Figure 2A, B). The impaired expression of SOD1 was confirmed in infant AT-MSCs with knockdown of SOD1 (SOD1kd) (Fig. 2B). Of note, the ROS level was increased, and cellular senescence was induced in infant AT-MSCs with SOD1kd, resulting in a decrease ability to decrease necrotic area in the transplanted mice (Fig. 2C–E). Similarly, the knockdown of SOD3 expression in infant AT-MSCs (Fig. 2F) resulted in enhanced ROS accumulation in these cells (Fig. 2G). Interestingly, in contrast to SOD1, the knockdown of SOD3 had no marked effect on the cellular senescence of infant AT-MSCs (Fig. 2H). However, the knockdown of SOD3 also resulted in the impaired ability to decrease necrotic area of infant AT-MSCs (Fig. 2I).

We next investigated the functions of SOD1 and SOD3 in the rescued ability of elderly AT-MSCs to decrease necrotic area by individually overexpressing SOD1 or SOD3 in elderly AT-MSCs and evaluating the subsequent effects. The up-regulation of SOD1 or SOD3 in elderly AT-MSCs was confirmed (Fig. 2J) and the characterization of AT-MSCs showed that elderly AT-MSCs with the up-regulation of SOD1 or SOD3 still maintained the proliferative ability and MSC marker profile (Supplementary Figure 2C, D). The individual overexpression of either SOD1 or SOD3 resulted in the decreased expression of ROS in elderly AT-MSCs (Fig. 2K); however, only the overexpression of SOD1 reduced the number of β-gal-positive cells which related to the cellular senescence of elderly AT-MSCs (Fig. 2L). Next, the effects of the overexpression of SOD1 or SOD3 on the healing functions of elderly AT-MSCs were examined by the gene expression profile and an in vivo transplantation study. The results showed that the expression of either SOD1 or SOD3 increased the expression of angiogenic factors and, consistently, improved the ability of elderly AT-MSCs to decrease necrotic area in diabetic mice compared to wild-type elderly AT-MSCs (Fig. 2M,N).

Taken together, these data showed the direct roles of SOD1 and SOD3 in the regulation of ROS, cellular senescence, and in vivo functions. Both SOD1 and SOD3 suppressed the accumulation of ROS and maintained the ability to decrease necrotic area; however, only SOD1 was found to play a role related to the cellular senescence of AT-MSCs. Therefore, these data suggested that, in AT-MSCs, SOD1 acts differently from SOD3, not only regulating the ROS balance but also involved in the cellular senescence process.

### The co-overexpression of SOD1 and SOD3 significantly improved the poor ability of elderly AT-MSCs to decrease necrotic area by the activation of the pERK/ERK pathway

**Figure 3:**
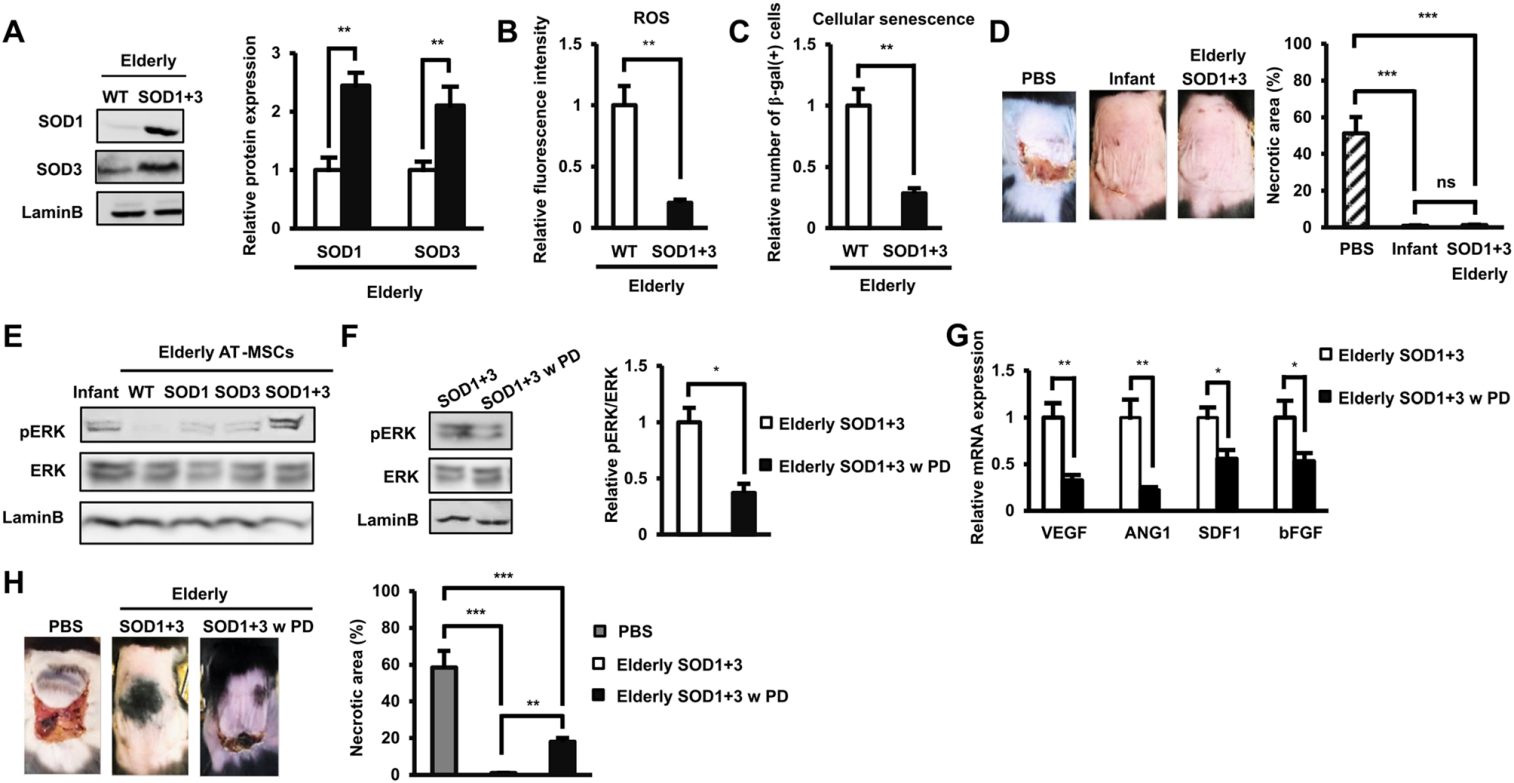
The co-overexpression of SOD1 and SOD3 significantly improved the poor functions of elderly AT-MSCs by the activation of the pERK/ERK pathway. (**A**) The protein expression of wild-type elderly AT-MSCs or with the co-overexpression of SOD1 and SOD3 (n = 3). (**B**) The ROS expression in wild-type elderly AT-MSCs or with the co-overexpression of SOD1 and SOD3 (n = 3). (**C**) The cellular senescence of wild-type elderly AT-MSCs or with the co-overexpression of SOD1 and SOD3 (n = 3). (**D**) Transplantation of wild-type elderly AT-MSCs or with the co-overexpression of SOD1 and SOD3 to an in vivo streptozotocin-induced diabetic ischemic flap mouse model (n = 3). (**E**) The protein expression of pERK/ERK in infant AT-MSC, wildtype elderly AT-MSCs, elderly AT-MSCs with the individual overexpression of SOD1 or SOD3 or elderly AT-MSCs with the co-overexpression of SOD1 and SOD3 (n = 3). (**F**) The protein expression of pERK/ERK under the presence of a MEK inhibitor (n = 3). (**G**) The mRNA expression of wound healing-related growth factors in elderly AT-MSCs with the co-overexpression of SOD1 and SOD3 under the presence of a MEK inhibitor (n = 3). (**H**) Transplantation of elderly AT-MSCs with the co-overexpression of SOD1 and SOD3 under the presence of a MEK inhibitor to an in vivo streptozotocin-induced diabetic ischemic flap mouse model (n = 3). In all above experiments, elderly AT-MSCs were derived from 3 different donors (n = 3). SOD1 + 3: elderly AT-MSCs with co-overexpression of SOD1 and SOD3. PD098059 (PD) was used as a MEK inhibitor. The data represent the mean ± SD. ***P < 0.001, **P < 0.01, *P < 0.05, *ns* no significance. The experiments were performed in triplicate. Full-length Western blots are presented in Supplementary Figure S4.

Since the overexpression of either SOD1 or SOD3 resulted to the partial improvement of the ability of elderly AT-MSCs to decrease necrotic area (Fig. 2N), we next examined the co-effects of the co-overexpression of both SOD1 and SOD3 in elderly AT-MSCs (Fig. 3A). First, co-overexpression of both SOD1 and SOD3 showed no significant alteration on the proliferative ability and MSC marker profile of elderly AT-MSCs (Supplementary Figure 2C, D). As expected, the co-overexpression of SOD1 and SOD3 significantly reduced the ROS level in elderly AT-MSCs and the number of β-gal-positive elderly AT-MSCs (Fig. 3B,C). Co-overexpression of SOD1 and SOD3 also reduced the expression of inflammatory cytokines in elderly AT-MSCs and enhanced the paracrine effects of elderly AT-MSCs on the angiogenic ability of EPC and EC (Supplementary Figure 2E, F). Furthermore, this co-overexpression fully rescued the ability of elderly AT-MSCs to decrease necrotic area in diabetic mice (Fig. 3D).

Next, we examined the ROS-mediated signaling pathways involved in the enhanced functions of SOD1/SOD3-overexpressing elderly AT-MSCs to decrease necrotic area in the flap mouse model. SOD1 reportedly promotes the phosphorylation of ERK1/2 in rat primary motor neurons^23^, while the overexpression of SOD3 induces the Ras-ERK pathways in rats^24^. Consistently, we found that the overexpression of both SOD1 and SOD3 caused the activation of the pERK/ERK pathways by the induced phosphorylation of ERK in elderly AT-MSCs (Fig. 3E). To examine whether or not the pERK/ERK pathway is involved in the detrimental effects of aging on the ability of AT-MSCs to decrease necrotic area of flap mouse model, we inhibited the MEK/ERK pathway in SOD1/SOD3-cooverexpressing elderly AT-MSCs using the MEK inhibitor PD098059 and observed the consequent effects on the cells. The impaired expression of phosphorylated ERK was confirmed (Fig. 3F), and the results showed that inhibition of the MEK/ERK pathway significantly down-regulated the expression of wound healing-related growth factors (Fig. 3G). Interestingly, treatment with a MEK inhibitor reversed the rescued functions of SOD1/SOD3-cooverexpressing elderly AT-MSCs, which partially resulted in the impaired ability of treated cells (Fig. 3H).

Taken together, these data suggested that the co-overexpression of SOD1 and SOD3, which had combined effects of dramatically reducing the ROS level and the number of β-gal-positive cells, resulted in the complete restoration of the ability of elderly AT-MSCs to decrease necrotic area in the streptozotocin-induced diabetic mice. In addition, aging-associated ROS impaired the ability of AT-MSCs to decrease necrotic area of flap mouse model by the inactivation of the MEK/ERK pathways.

### Rejuvenation of elderly AT-MSCs with infant AT-MSC-derived EVs by inhibiting the accumulation of ROS

**Figure 4:**
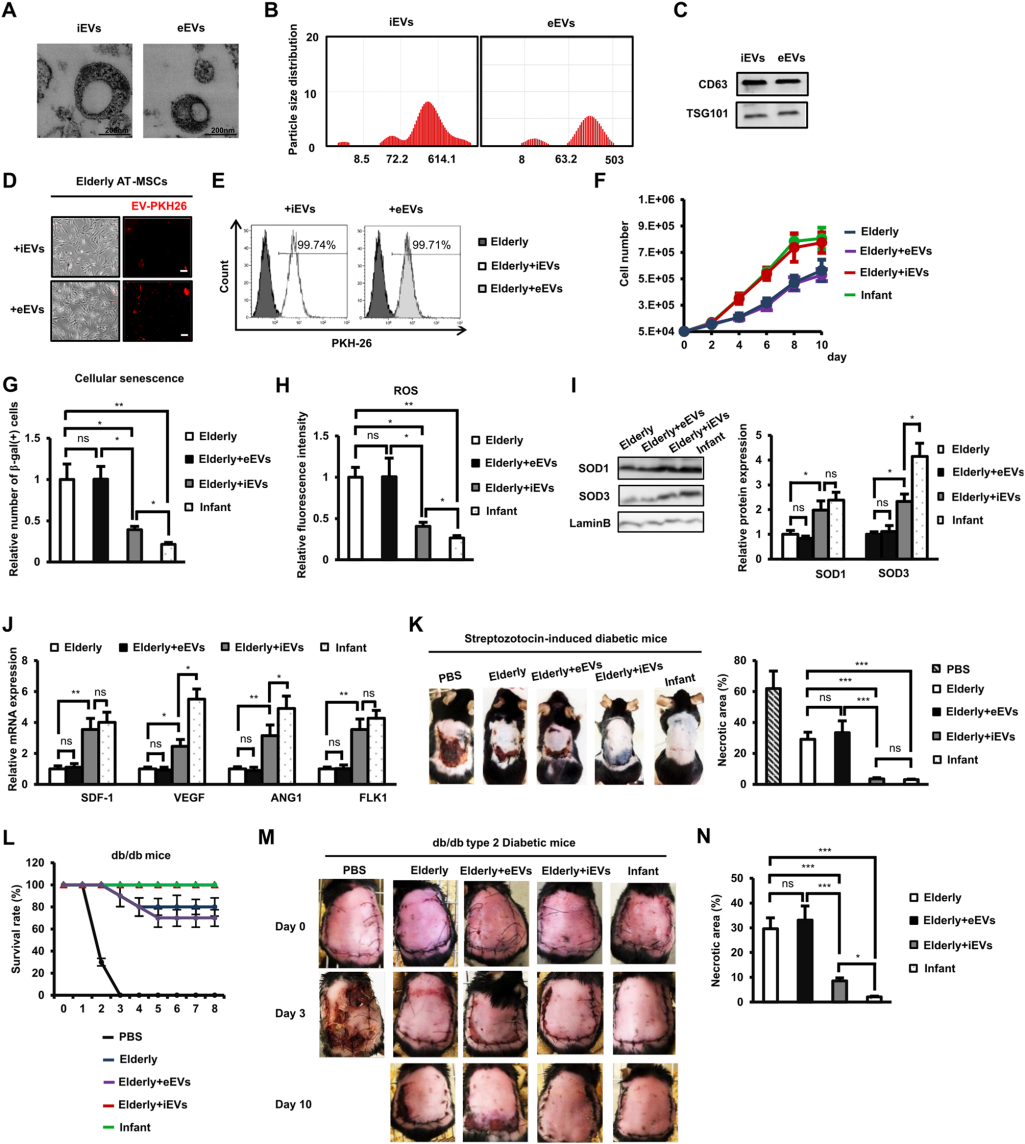
Rejuvenation of elderly AT-MSCs with infant AT-MSC-derived extracellular vesicles by inhibiting the accumulation of ROS. (**A**) The analysis of EVs under the transmission electron miscroscope. Bars indicated 200 nm (n = 5). (**B**) The particle size of EVs examined by a fiberoptic dynamic light scattering photometer (FDLS-3000) (n = 5). (**C**) The marker expression of EVs examined by Western blotting (n = 5). (**D**) The incorporation of PKH26-labeled EVs into elderly AT-MSCs, magnification 4 ×. Bar indicates 200 μM (n = 5). (**E**) The incorporating efficiency examined by a FACS analysis (n = 5). (**F**) The growth curve of AT-MSCs (n = 5). (**G**) The cellular senescence of elderly AT-MSCs with EV incorporation (n = 5). (**H**) The ROS expression of elderly AT-MSCs with EV incorporation (n = 5). (**I**) The SOD1 and SOD3 protein expression of elderly AT-MSCs with EV incorporation (n = 5). (**J**) The mRNA expression of growth factors in elderly AT-MSCs with EV incorporation (n = 5). (**K**) The transplantation study of elderly AT-MSCs with EV incorporation in streptozotocin-induced diabetic mice (n = 5). (**L**) The survival rate of db/db mice transplanted with AT-MSCs (n = 5). (**M**) The necrotic area of db/db mice transplanted with AT-MSCs. (**N**) Quantification of the necrotic area of db/db mice on day 10 post-transplantation with AT-MSCs (n = 5). In all above experiments, infant AT-MSCs and elderly AT-MSCs were derived from 5 different donors, respectively (n = 5). AT-MSCs were at passage 3 to passage 8, and the comparison was conducted with infant AT-MSCs and elderly AT-MSCs at the same passage number. EVs were isolated from infant AT-MSCs or elderly AT-MSCs from passage 4 to passage 6. The data represent the mean ± SD. ***P < 0.001, **P < 0.01, *P < 0.05, *ns* no significance. The experiments were performed in triplicate. Full-length Western blots are presented in Supplementary Figure S5.

We previously reported the ability of EVs derived from healthy donor AT-MSCs to rescue the impaired functions of AT-MSCs in type 2 diabetic patients^25^. This finding suggested that EVs that contain miRNA, proteins, and lipids from the parental cells are effective tools of gene transfer to modify the characteristics and functions of target cells. Therefore, in the present study, we explored whether or not infant AT-MSC-derived EVs (iEVs) could rejuvenate and improve the functions of elderly AT-MSCs. To answer this question, we isolated and incorporated either iEVs or eEVs into other elderly AT-MSCs and analyzed the functions of these EV-incorporated cells. The Transmission electron microscopy analysis clearly shows that both iEVs and eEVs are oval particles from 60 to 500 nm in diameter (Fig. 4A) which is consistent with previous study^26^. In addition, the analysis of EVs sizes using a Particle Size Analyzer showed that both iEV and eEV fractions included the combination of exosomes and microvesicles, as indicated by particle sizes of 60–600 nm (Fig. 4B), expressing typical markers of CD63 and TSG101 (Fig. 4C). The incorporation of EVs by elderly AT-MSCs confirmed that 100% of the target elderly AT-MSCs in the population contained either iEVs or heterogeneous eEVs according to the observation of PKH26-labeled EV signals under the microscope (Fig. 4D) and a fluorescence-activated cell sorting (FACS) analysis (Fig. 4E) before the functional analysis of MV-incorporated elderly AT-MSCs.

The data showed that while eEVs showed no effects, iEVs significantly increased the proliferation of target elderly AT-MSCs, resulting in values as high as those in infant cells (Fig. 4F). In addition, iEVs also reduced the number of β-gal-positive elderly AT-MSCs (2.56-fold decrease in iEV-incorporated elderly AT-MSCs compared to those without iEVs, Fig. 4G). Interestingly, the incorporation of iEVs significantly reduced the accumulation of intracellular ROS in elderly AT-MSCs (2.5-fold decrease, Fig. 4H), possibly due to the upregulation of SOD1 and SOD3 protein expression (SOD1: 1.97-fold increase, SOD3: 2.32-fold increase, Fig. 4I). In addition, the incorporation of iEVs upregulated the expression of wound healing-related cytokines (*sdf1*,* vegf*,* ang1*, and *flk1*) in elderly AT-MSCs, while the incorporation of eEVs showed no marked effect on the expression profile (Fig. 4J).

We next examined the effects of iEVs on the in vivo functions of elderly AT-MSCs. Transplantation studies showed that, after incorporating iEVs, elderly AT-MSCs displayed a significantly ability to decrease the necrotic area in flap mice, similar to that by infant AT-MSCs (necrotic area of mice on day 7 transplanted with elderly AT-MSCs: 29.135%, with elderly AT-MSCs incorporated with eEVs: 33.34%, with elderly AT-MSCs incorporated with iEVs: 3.53%, and with infant AT-MSCs: 3.045%, n = 5, *p* < 0.001, Fig. 4K). To determine the most effective therapy for diabetic foot ulcer, a common complication of type 2 DM (T2DM), we next performed a transplantation study using db/db mice as a T2DM model. As a result, mice injected with PBS showed severe inflammation and died at two to three days post-surgery (Fig. 4L,M). Although transplantation with elderly AT-MSCs improved the survival of db/db mice with wounds (80% db/db mice survived to day 8 post-transplantation; Fig. 4L,M), a substantial necrotic area remained. Of note, transplantation with either infant AT-MSCs or iEV-incorporated elderly AT-MSCs significantly decreased necrotic area in mice (necrotic area of mice on day 10 transplanted with elderly AT-MSCs: 29.135%, with elderly AT-MSCs incorporated with eEVs: 33.12%, with elderly AT-MSCs incorporated with iEVs: 8.57%, with infant AT-MSCs: 2.12%, Fig. 4N).

Taken together, these data suggested the role of iEVs in rejuvenating elderly AT-MSCs by inducing the proliferation and upregulating the impaired cytokine expression, thereby promoting the functions of elderly AT-MSCs to decrease necrotic area in both type 1 and type 2 diabetic mice.

## Discussion

**Figure 5:**
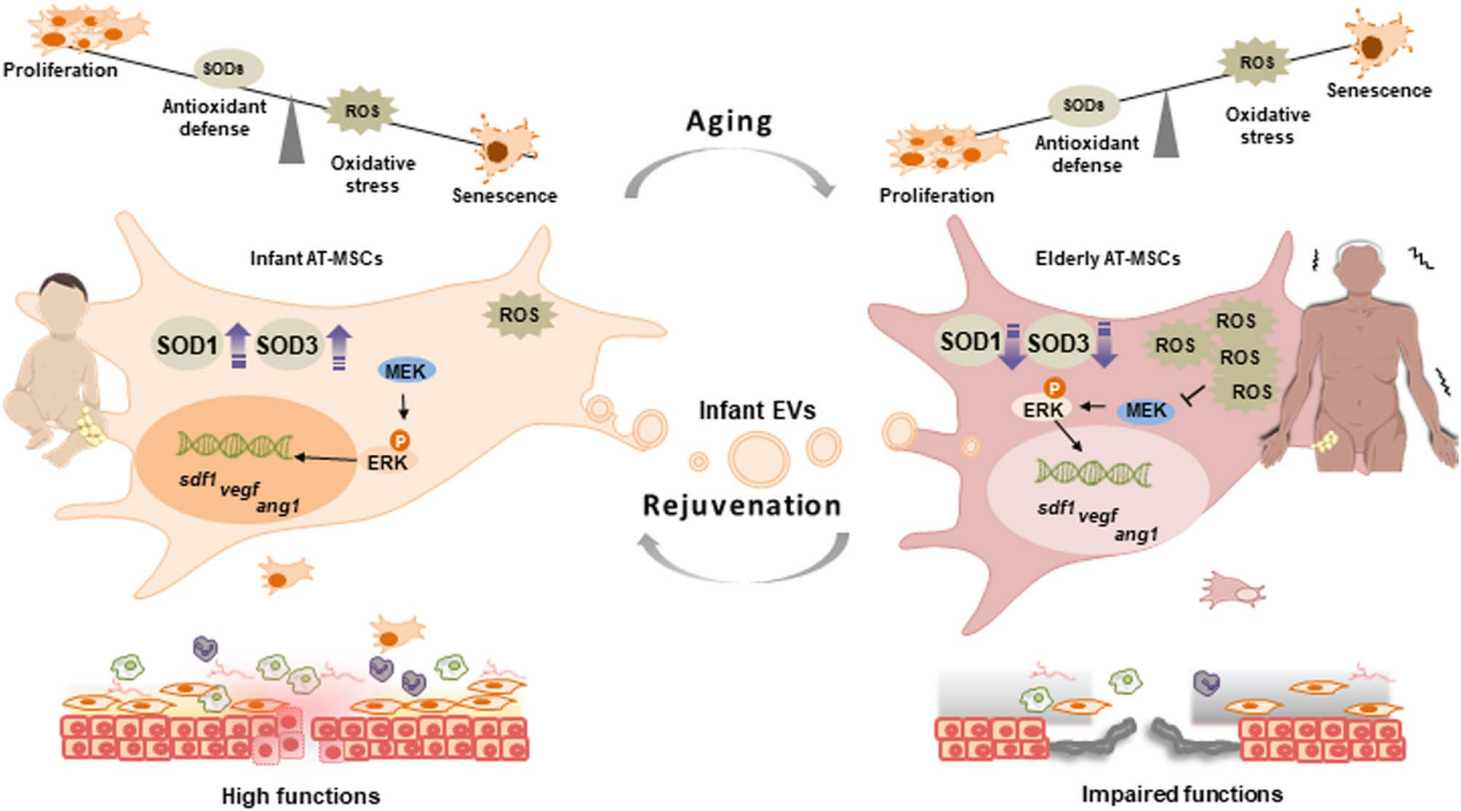
Proposed model: Infant extracellular vesicles rejuvenated elderly AT-MSCs by inhibiting the elevation of ROS. Elderly-derived AT-MSCs showed ROS accumulation due to the impairment of antioxidant enzymes, including SOD1 and SOD3, resulting in the suppression of the MEK/ERK pathway, which is involved in the function to decrease necrotic area. The incorporation of infant AT-MSC-derived EVs rejuvenated the elderly AT-MSCs by promoting the proliferation and expression of SOD1 and SOD3 and inhibiting the elevation of ROS levels and cellular senescence, thus resulting in improved functions of elderly AT-MSCs to decrease necrotic area.

Substantial evidence of the aging-associated reduction in the functions of adult stem cells prompted us to develop novel strategies for rejuvenating elderly stem cells for anti-aging research and therapies^16^. In the present study, we examined the correlation of the increased ROS levels and impaired cellular functions of elderly AT-MSCs as well as the rejuvenation of AT-MSCs by infant EVs. Indeed, we found a significant increase in ROS due to the impairment of SOD1 and SOD3 antioxidant enzymes in elderly AT-MSCs compared with infant AT-MSCs. Consequently, the impairment of SOD1 and SOD3 directly caused a reduction in the paracrine effects of elderly AT-MSCs on EPC and EC functions, and the ability of elderly AT-MSCs to decrease necrotic area of the flap mouse model. Furthermore, while both SOD1 and SOD3 were responsible for the elevated intracellular ROS levels, only SOD1 showed an effect on the number of β-gal-positive cells which related to the cellular senescence of elderly AT-MSCs. The co-overexpression of SOD1 and SOD3 suppressed the accumulation of intracellular ROS and cellular senescence, which restored the impaired functions of elderly AT-MSCs by the activation of the MEK/ERK pathways. Of note, the incorporation of infant AT-MSC-derived EVs recovered the aging-associated impairment of elderly AT-MScs by promoting proliferation, reduced the number of β-gal-positive cells, and inhibiting the elevation of ROS by upregulating the expression of SOD1 and SOD3. Consequently, infant EVs induced the ability of elderly AT-MSCs to decrease necrotic area in both type 1 and type 2 diabetic mice (Fig. 5).

A previous study showed a significant increase in wound healing in older mice transplanted with younger donor mouse-derived bone marrow MSCs^27^, suggesting the correlation of the aging and wound-healing ability of MSCs. In addition, the accumulation of ROS during aging has been suggested to be involved in the reduced functions of stem cells^28,29^. In the present study, we showed that, during the aging process, human AT-MSCs exhibit the impairment of SOD1 and SOD3 which were involved in the increased intracellular ROS levels, the altered expression of pro-inflammatory factors and cytokines related to wound healing^30^. Consequently, compared to infant AT-MSCs, elderly AT-MSCs showed the impaired paracrine effects to support the angiogenic functions of EPCs and ECs and the ability to reduce necrotic area of the flap diabetic mouse model, suggesting the need for the modification of elderly AT-MSCs before their clinical use. However, it is worth to note that in the present study, we used H2DCFDA as a ROS detection which signal depends on various cell parameters, such as acidity, catalytic activity, and cell size. In addition, it is necessary to further clarify these altered paracrine effects on not only EPCs and ECs but other cell types contributing to the wound-healing process as well.

To examine the role of ROS in the functions of elderly AT-MSCs, we first examined the effects of pharmacological intervention with antioxidants on elderly AT-MSCs. Although the treatment with NAC reduced the ROS level of elderly AT-MSCs, no effect on the in vivo therapeutic potential of the cells was observed, suggesting aging impairs the functions of AT-MSCs not only by the elevation of ROS. Meanwhile, treatment with Edaravone which reduced both the ROS level and the number of β-gal-positive cells resulted in increased in vivo functions of elderly AT-MSCs. Edaravone, initially developed as an intravenous treatment of acute ischemic stroke, is a free radical scavenger that reduces oxidative stress^31^. A previous report showed that treatment with Edaravone protects mouse-derived bone marrow MSCs against hypoxia by inhibiting the intracellular accumulation of ROS^32^. In addition, transplantation of Edaravone-treated MSC promotes angiogenesis and resident cardiac stem cell’s regeneration in a rat model of myocardial infraction^32^. Furthermore, Edaravone treatment of human umbilical cord-derived MSCs restored the levels of endogenous antioxidant enzymes (particularly SOD1) impaired by oxidative stress and improved the hepatic functions^33^. In our study, the Edaravone-treated elderly AT-MSCs showed decreased ROS levels and reduced number of β-gal-positive cells, resulting in the recovery of elderly AT-MSCs’ in vivo functions. Because the number of β-gal-positive cells is a marker of cellular senescence which has been suggested to be associated with MSCs’ dysfunctions in aging process, we hypothesized that beside ROS level, cellular senescence is also involved in the impaired functions of elderly AT-MSCs.

In the regulation of ROS in mammals, superoxide dismutases (SODs) are the major antioxidant defense enzymes that catalyze the conversion of O_2_^·−^ and effectively protect cells against oxidative stress^34^, prevent mitochondrial dysfunction, and function in cellular signaling^34^. The SOD family includes three isoforms: the cytoplasmic Cu/ZnSOD (SOD1), the mitochondrial MnSOD (SOD2), and the extracellular Cu/ZnSOD (SOD3), which are the products of different genes but catalyzes the same reaction^35^. A previous report showed that the age-associated deficiency of SOD1 causes dysfunction in wound healing of dermal fibroblasts^36^. In the present study, we found that aging impaired the expression of SOD1 and SOD3, with no significant effects on SOD2 in AT-MSCs. Genetic comparisons indicate that similarities exist between the *sod1* and *sod3* genes at certain levels of amino acid homology, while *sod2* does not share any substantial amino acid homology with either *sod1* or *sod3*^37^. In addition, the SOD1 and SOD3 active sites are very similar to each other and exhibit Cu-dependent specific activity^37^. Given these findings, future studies should clarify how to regulate the SOD1 and SOD3 expression in AT-MSCs. In addition, beside the regulation of ROS, it is worth to note while SOD1 was associated with the expression of β-gal-positive in AT-MSCs, the role of remains unclear SOD3 in other aging phenotypes of AT-MSCs. Because the overexpression of SOD3 enhanced the functions of elderly AT-MSCs, it is speculated that complex mechanisms might exist underlying the impaired functions of AT-MSCs by aging, which not only limit at ROS level and cellular senescence.

While we examined the co-effects of the knockdown of both SOD1 and SOD3 in infant AT-MSCs, the impaired expression of both SOD1 and SOD3 resulted in diminished cell proliferation (Supplementary Figure 2A). This result suggested that both SOD1 and SOD3 contribute to the cellular survival and proliferation. The role of SOD1 as a guardian of most cellular structures, including genomic DNA, has been reported in numerous reports^38–40^. In addition, a previous report showed that SOD3 promotes the MSC survival through induced autophagy and promotes FoxO3a trafficking to the nucleus^41^. Of note, the co-overexpression of SOD1 and SOD3 in elderly AT-MSCs dramatically suppressed the level of ROS and number of β-gal-positive cells, thereby enhancing the cellular functions in our study. Consistent with previous studies^23,24^, our study also showed that the overexpression of SOD1 and SOD3 modulated the functions of AT-MSCs by activating the MEK/ERK pathways. Interestingly, using a MEK inhibitor, the functions of elderly AT-MSCs restored by SOD1 and SOD3 were eliminated, suggesting that this pathway is the direct target of ROS in the regulation of the function of AT-MSCs to decrease necrotic area of the flap mouse model. Further studies are needed in order to clarify the downstream key factors of the MEK/ERK pathway, which is regulated by ROS in AT-MSCs during aging.

While genetic modification and pharmacological intervention might help to rejuvenate elderly AT-MSCs by affecting several aging phenotypes including the ROS level, the β-gal-expression and the therapeutic functions, the risk of side effects and other safety issues are still a matter of concern^16,42^. Of note, it has been reported the aging of stem cells is induced by signals from the local microenvironment^2^, and the exposure of aged mouse skeletal muscle to a young systemic serum environment promoted the proliferation and activity of aged cells^5^ which suggested the idea in the present study that EVs derived from infant AT-MSCs might appear to be promising candidates for modifying the target cells. Indeed, the addition of infant AT-MSC-EVs to the culture of elderly AT-MSCs significantly upregulated the antioxidant defense, thus inhibiting the elevation of ROS and reduced the number of β-gal-positive cells, and promoting the proliferation. This resulted in the improvement of the functions of the aged cells to decrease necrotic area in the type 1 and 2 diabetic mouse models. These results provide novel insight into the ability of infant EVs to rescue the cellular functions impaired by aging. It has been reported that EVs modified the phenotypes of recipient cells by several mechanisms such as: loading their mRNA and proteins which plays the functions in recipient cells and transferring their miRNA, lncRNA, or other non-coding RNA to regulate the expression of local genes in recipient cells^43^. In our study, at the same amount of total protein of EVs, we examined that iEVs showed the higher expression of angiogenic proteins such as VEGF, ANG1, and FLK1 (Supplementary Figure 2G). These data suggested the different content of iEVs and eEVs and that iEVs might load their angiogenic proteins to the elderly AT-MSCs, which might be involved in the improved functions of elderly AT-MSCs to decrease the necrotic area of the flap mouse model. It is worth for a further comparison of content of iEVs and eEVs, such as mRNA, miRNA, to clarify underlying mechanisms related to the ability of infant EVs to change the phenotypes of elderly cells.

Notably, our present study only limited at the ability of infant EVs to reduce ROS and correct several effects which accompany cellular senescence such as impaired proliferation, declined in vivo functions to reduce the necrotic area, and increased number of β-gal-positive cells. Because aging phenotypes of MSCs includes a complex of mechanisms such as telomere attrition, DNA damage and mutation, and mitochondrial functions^42^, whether infant EVs allow reversing the aging process of elderly AT-MSCs requires the further analysis of the complete set of aging phenotypes of MSCs.

In summary, the present study demonstrated that the age of donors is correlated with the redox balance, cellular senescence, and the functions of AT-MSCs to decrease necrosis in the skin flap model. In particular, aging impaired the functions of AT-MSCs through an increased ROS level, resulting in the reduced expression of both SOD1 and SOD3 antioxidant enzymes. Notably, using EVs derived from infant AT-MSCs can be useful for the improvement of elderly stem cell functions by inhibiting ROS elevation.

## Materials and methods

### Statement

All experiments and methods were carried out in accordance with the relevant guidelines and regulations according to the amended Declaration of Helsinki. All of the experiments related to human samples were approved by the Ethics Committee of the University of Tsukuba. Human samples collection, including adipose tissues and umbilical cord blood, and relevant experiments were performed after obtaining informed consent from all donors. For the adipose tissues derived from infant subjects, the collection and all related experiments were conducted after obtaining the informed consent from the parents and/or legal guardians. In addition, all animal experiments were approved by the Animal Care Committee of the University of Tsukuba.

### Isolation and culture of AT-MSCs and endothelial progenitor cells (EPCs)

Human adipose tissues were collected from the infants (n = 5, 1–12 months) and elderly donors (n = 5, 70–80 years old) who are undergoing the treatment in Department of Cardiovascular Surgery, University of Tsukuba Hospital, Tsukuba, Japan. AT-MSCs were isolated according to a previously described method^44^. In brief, adipose tissues were minced and digested with 0.1% collagenase (Invitrogen, Waltham, Massachusetts, CA, USA) in phosphate-buffered saline (PBS) at 37 °C for 1 h. AT-MSCs were harvested by the centrifugation at 1600 rpm in 7 min, then suspended in the culture medium (Iscove’s Modified Dulbecco Medium [IMDM]; Invitrogen), containing 10% heat-inactivated fetal bovine serum (FBS; Invitrogen), 2 mg/ml L-glutamine (Invitrogen), 100 units/ml penicillin (Invitrogen), and 5 ng/ml bFGF (Peprotech, Cranbury, NJ, USA). AT-MSCs were maintained and cultured at 37 °C in a 5% CO_2_ atmosphere. Culture medium was changed every 3 days. The AT-MSCs used for all experiments in this study were derived from passage 3 to passage 8.

Human umbilical cord blood samples were obtained from Department of Obstetrics and Gynecology, University of Tsukuba Hospital, Tsukuba, Japan and EPCs was isolated according to previously described method^45^. Then the hematopoietic cells and mononuclear cells were separated from the cord blood by density gradient centrifugation. After that, the pellet was suspended in the cultured medium in IMDM (Invitrogen), containing 10% heat-inactivated FBS; Invitrogen), 2 mg/ml l-glutamine (Invitrogen), 100 units/ml penicillin (Invitrogen), and 5 ng/ml bFGF (Peprotech) and cultured for 7 days. EPCs was isolated by the cell sorting to collect CD45-/ Dil-Ac-LDL+/CD31+ cells and cultured in the same culture medium. Medium was changed every three days. Cells were maintained and cultured at 37 °C in a 5% CO_2_ atmosphere.

### Collection of AT-MSC-derived conditioned medium (CM) and EVs

To collect the AT-MSC-CM and EVs, AT-MSCs derived from passage 4 to passage 6 were used. AT-MSCs were seeded at 10^6^ cells and cultured for 12 h. The culture medium was replaced with fresh IMDM containing 0.25% FBS, and incubation was continued for a further 48 h. The AT-MSC-derived CM was then collected by centrifuging at 1000 rpm for 5 min followed by centrifugation at 2100 rpm for 20 min to remove the cell debris. For AT-MSC-EV isolation, the AT-MSC-CM was ultracentrifuged at 37,000 rpm for 70 min at 4 °C. The pellet was then resuspended in PBS, and the protein concentration was examined by a Bradford assay. In addition, the morphology of EVs were analyzed using the transmission electron microscopy and the size of the EVs was measured using a Particle Size Analyzer (FDLS3000; Shimadzu Corporation, Kyoto, Japan), and the EV-surface markers were examined by the Western blotting with anti-CD63 and anti-TSG101 antibodies (Table 2).

### Establishment of sod1kd infant AT-MSCs, sod3kd infant AT-MSCs and sod1 + 3kd infant AT-MSCs

Mission shRNA lentiviral particles of *sod1* or *sod3* (Sigma Aldrich, Saint Louis, MO, USA) were used to establish the infant AT-MSCs with knockdown of SOD1 or SOD3 and the protocols were conducted as the instruction from the manufacturer. Scrambled control transduction particles (Sigma Aldrich) were used as the control. Briefly, infant AT-MSCs were plated in complete culture medium until reaching 60% confluency. Mission shRNA lentiviral particles of *sod1* or *sod3* were thaw slowly on ice and gently added to the culture contained infant AT-MSCs at a MOI 5. The cell-viral particle mixture was incubated at 37 °C overnight. On the next day, the viral particle-containing medium was removed and replaced with fresh complete culture medium containing 4 μg/ml puromycin. The medium was replaced every 3 days until resistant colonies were identified. The effect of shRNA of SOD1 or SOD3 was assessed by qRT-PCR and Western Blot analysis.

In order to establish the infant AT-MSCs with knockdown both SOD1 and SOD3, the infant AT-MSCs with knockdown of SOD1 was used for the transduction of mission shRNA lentiviral particles of *sod3* and the effect was examined by qRT-PCR and Western Blot analysis.

### Establishment of elderly AT-MSCs with the overexpression of SOD1, SOD3, or SOD1 and SOD3

To establish the elderly AT-MSCs with overexpression of SOD1 or SOD3, MSCV-hSOD1-IRESS-EFGP or MSCV-hSOD3-IRESS-EFGP was constructed and transfected into HEK cells using Lipofectamine LTX and PLUS reagent (Life Technologies). After 48 h of transfection, the GFP signal was confirmed and the virus-contained medium was collected and transfected to PT67 cells. At 48 h after transfection, the GFP-positive PT67 cells were sorted and expanded to produce the stable virus production cell line. The virus-containing medium collected from the culture of GFP-positive PT67 cells was used for transfection to elderly AT-MSCs. After 48 h of transfection, the cells’ GFP signal was observed and the cells were expanded. The GFP-positive elderly AT-MSCs were then sorted and continued to culture for the subsequent experiments. The effects of overexpression of SOD1 or SOD3 were examined by qRT-PCR and Western Blot analysis.

To establish the elderly AT-MSCs with co-overexpression of SOD1 and SOD3, the elderly AT-MSCs with overexpression of SOD1 was used for the transfection of virus containing MSCV-hSOD3-IRESS-EFGP. The effects of co-overexpression of SOD1 and SOD3 were examined by qRT-PCR and Western Blot analysis.

### Treatment of elderly AT-MSCs with antioxidants

In order to examine the effective concentration of Edaravone or NAC on elderly AT-MSCs, elderly AT-MSCs were placed at 10^3^/well in 96-well plate until reaching 50% confluency. Then elderly AT-MSCs were treated with Edaravone (Tocris bioscience, Bristol, UK) at the concentration range of 0, 5, 10, 20, 30, 40 µM or NAC (Sigma Aldrich) at the concentration range of 0, 0.5, 1, 2, 4, 8 mM. The effective concentration of Edaravone or NAC was examined by the cell viability and ROS expression every 6 h within 48 h.

For the analysis of effects of ROS on elderly AT-MSCs, elderly AT-MSCs were placed at 5 × 10^5^ cells in 10 cm-dishes and cultured under complete culture medium for 4 days until reaching 50% confluency. Then elderly AT-MSCs were treated with Edaravone with the effective concentration examined at 20 µM or NAC with the effective concentration examined at 2 mM for 24 h. Then the effects of antioxidants on the expression of ROS and functions of eldelry AT-MSCs were examined.

### Measurement of the intracellular ROS level

In order to measure the intracellular ROS level, 2′,7′-dichlorodihydrofluorescein diacetate (H2-DCFDA; Invitrogen) was used as the probe according to a previously described method^46^. In brief, after reaching 80% confluency, the cells were washed with PBS and incubated with PBS containing 10 μM DCFDA at 37 °C for 30 min. The fluorescence intensity was measured by the absorbance at 495 and 525 nm wavelengths using a photoemission spectrophotometer (Corona Electric Co., Ltd, Ibaraki, Japan).

### Reverse transcription–polymerase chain reaction (RT–PCR) and quantitative real-time PCR (qRT-PCR)

Total RNA from AT-MSCs was isolated using Sepasol-RNA I Super G (Nacalai Testque, Kyoto, Japan) according to the previous described method^44^. In order to synthesize cDNA, a RT-PCR kit (Toyobo Co., Ltd, Osaka, Japan) was used with 1 µg of total RNA. Next, the expression of the target genes was examined by quantitative PCR of cDNA. The reaction mixtures for qPCR were prepared using SYBRGreen Realtime PCR mastermix (Toyobo) and analyzed using a GeneAmp 7500Fast Realtime PCR System (Life Technologies). The sequences of primers used for the PCR reactions are listed in Table 1. The experiments were performed in triplicate, and data were calculated by the double-delta cycle number of thresholds (ΔΔCt) method.

**Table 1:** Primer sets used for quantitative PCR.

Gene	Forward primer	Reverse primer
*il6*	ACAAGAGTAACATGTGTGAAAGCAG	TATACCTCAAACTCCAAAAGACCAG
*il8*	TGCTTCCCCTTAGCATTTTGT	TGTCCAGCTATGCTAAAGTGC
*ccl5*	GAGGATTCCTGCAGAGGATCAAGACAG	TCCAAAGAGTTGATGTACTCCCGAACC
*ccl3*	CAGCCTGTGTAGGCAGTCAT	CTCCCCATCTCTCCCAATTTCC
*vegf*	AGATGAGCTTCCTACAGCACAAC	AGGACTTATACCGGGATTTCTTG
*ang1*	GCCTGATCTTACACGGTGCT	GGCCACAAGCATCAAACCAC
*sdf1*	AGAGCCAACGTCAAGCATCT	CTTTAGCTTCGGGTCAATGC
*bfgf*	AGAGCGACCCTCACATCAAGCTACAAC	ATAGCTTTCTGCCCAGGTCCTGTTTTG
*flk1*	AGTGTGGAGGACTTCCAGGGAGGAAAT	GGCCAAGCTTGTACCATGTGAGGTTCT
*β-actin*	GTGCGTGACATTAAGGAGAAGCTGTGC	GTACTTGCGCTCCAGGAGGAGCAATGAT

### Treatment of AT-MSCs with PD098059 (PD) as a MEK inhibitor

The treatment of AT-MSCs with PD098059 (P215, Sigma-Alrich) was conducted as the instruction of manufacturer and our previously reported^47^. Briefly, AT-MSCs were cultured until reaching 80% of confluency, then treated with PD at a concentration of 50 µM for 60 min. The effects of PD on the phosphorylation of ERK1/2 were examined by Western Blot analysis.

### Western blotting

AT-MSCs were collected, and nuclear protein was extracted according to the manufacturer’s protocol (Life Technologies) and previously described method^46^. The extracted protein concentration was measured by Bradford method (Bio-rad, CA, US). For the examination of protein expression in EVs, 10 µg of either iEVs or eEVs was mixed with an equal volume of Ripa buffer (Nacalai Testque), incubated on ice for 15 min, vortexed every 5 min. After that, the protein was mixed with sodium dodecyl sulfate (SDS) loading buffer (Wako Pure Chemical Industries, Ltd., Osaka, Japan) and heated at 95 °C for 3 min. Equal amounts of protein were separated through SDS–polyacrylamide gels and transferred onto PVDF membranes (EMD Millipore, Darmstadt, Germany) for Western blotting. The membranes were immunoblotted with primary antibodies which listed in Table 2. Horseradish peroxidase (HRP)-conjugated rabbit anti-goat IgG (Invitrogen), and HRP-conjugated goat anti-rabbit IgG (Invitrogen) were used as the secondary antibodies. The signals were detected by incubating the membrane with an enhanced chemiluminescence HRP substrate (EMD Millipore) for 1 min and visualized using an Image Quant LAS 4000 System (GE Healthcare Life Sciences, Marlborough, MA, USA).

**Table 2:** Primary antibodies used for Western blotting.

Antibody	Source	Company	Dilution rate
Human SOD1 Antibody (AF3418)	Polyclonal Goat IgG	R&D System, Minneapolis, MN, USA	1:1000
Human SOD2 Antibody (AF3419)	Polyclonal Goat IgG	R&D System	1:200
Human SOD3 Antibody (AF3420)	Polyclonal Goat IgG	R&D System	1:1000
Human GPX1 Antibody (AF3798)	Polyclonal Goat IgG	R&D System	1:200
Human Catalase Antibody (MAB3398)	Polyclonal Goat IgG	R&D System	1:1000
Human Lamin B Antibody (sc-6216)	Polyclonal Goat IgG	Santa Cruz Biotechnology	1:1000
Human Phospho-p44/42 MAPK (ERK) Antibody (9101S)	Polyclonal Rabbit IgG	Cell Signaling Technology, Danvers, MA, USA	1:1000
Human p44/42 MAPK (ERK) Antibody (9102S)	Polyclonal Rabbit IgG	Cell Signaling Technology	1:1000
Human CD63 Antibody (CSB-PA006039)	Polyclonal Rabbit IgG	Cusabio Technology LLC, Houston, TX, USA	1:1000
Human TSG101 Antibody (CSB-PA060017)	Polyclonal Rabbit IgG	Cusabio Technology LLC	1:1000
Human VEGF Antibody (sc-507)	Polyclonal Rabbit IgG	Santa Cruz Biotechnology	1:1000
Human ANG1 Antibody (sc-6320)	Polyclonal Goat IgG	Santa Cruz Biotechnology	1:1000
Human FLK1 Antibody (sc-315)	Polyclonal Rabbit IgG	Santa Cruz Biotechnology	1:1000

### Scratch assay

AT-MSCs were seeded at 0.75 × 10^5^ cells/well in 4-well plates and cultured at 37 °C under 5% CO_2_ for 24 h. The cells were then treated with 10 µg/mL mitomycin-C in culture medium and incubated for 3 h before being scratched using a 1-mm pipette tip. AT-MSCs were washed with 1 × PBS and incubated at 37 °C under 5% CO_2_ for 24 h and observed under a microscope at 4 × objective lenses. Pictures were taken at 0, 12, 16, and 24 h. The un-migrated areas were analyzed using the ImageJ software program (Vetsion 1.51, an open source image processing software, National Institute of Mental Health, Bethesda, MD, USA)^48^ and calculated to give a % migrated area at 0 and 24 h.

### Co-culture of AT-MSCs and EPCs

AT-MSCs were seeded at 5 × 10^4^ cells in the lower chamber of an 8-μm pore transwell (Corning Incorporated, New York, NY, USA) containing 500 μl completed culture IMDM. Cells were maintained at 37 °C in a 5% CO_2_ atmosphere for 24 h to allow cell attachment. After 24-h incubation, the medium was replaced with 500 μl IMDM supplemented with 2% (v/v) FBS and 100 U/ml penicillin–streptomycin, and then cells were cultured for a further 24 h. On the following day, 4 × 10^4^ EPCs were seeded into the upper chamber of the transwell. Coculture was maintained at 37 °C in a 5% CO_2_ atmosphere for a further 6 h. At the end of culture, the number of EPCs that had migrated to the other side of the transwell inserts was determined by hematoxylin staining and counted. The EPCs cultured in transwells whose lower chamber contained cell-free medium were used as controls.

### The tube formation assay

In brief, 4-well plates were coated with growth factor-reduced matrigel (BD Biosciences, San Jose, CA, USA) and then incubated at 37 °C for 30 min. EPCs were then seeded at a density of 5 × 10^4^ cells to the Matrigel-coated wells and cultured at 37 °C. After 9 h, the tubes formed in 10 randomly selected fields per well were counted, and the average number was determined using the ImageJ software program (NIH)^48^.

### The cellular senescence assay

AT-MSCs were seeded at 10^4^ cells/well in a 96-well plate and incubated overnight. The following day, the cells were washed with PBS and treated with 50 µl β-glo assay reagent (Promega, Madison, WI, USA) and 50 µl PBS according to the manufacturer’s protocol. Cells were incubated for 30 min at room temperature, following by the measurement of the absorbance using a luminescence microplate reader (Thermo Fisher Scientific, Waltham, MA, USA).

### Ischemic flap mouse model

Male C57BL/6 and db/db mice were purchased from Charles River Japan, Inc., Yokohama, Japan), given food and water ad libitum, and maintained in a 12-h light/dark cycle in the Animal Research Center of the University of Tsukuba. All animal experiments were approved by the Animal Care Committee of the University of Tsukuba. Eight-week-old mice were used in the experiments. Streptozocin-induced type 1 diabetic mice were developed as described previously^49^. In brief, C57BL/6 mice were subjected to continuous intraperitoneal injection of streptozotocin (40 mg/kg) for 5 days. One week after injection, the blood glucose was confirmed to be higher than 11.1 mmol/l.

The ischemic flap mouse model was developed as described previously^50^. The mice were anesthetized using avertin, and a skin incision (3 × 2 cm) was made to create an ischemia gradient. The mice were divided into 3 groups: no operation (n = 5); sham injection with PBS (n = 5); and experimental group with injection of AT-MSCs derived from infants or elderly subjects (n = 15, respectively). AT-MSCs were injected at 0.5 × 10^6^ cells/mouse at 4 points in the skin flap. Immunosuppression was induced by the intraperitoneal injection of cyclosporin A (20 mg/kg; Wako) every 2 days. Observation was performed every day. For the histological analyses, flap tissues were collected at two different time points: on day 3 post-transplantation for the study of inflammatory cell recruitment and on day 7 post-transplantation for the study of neovascularization. The images of the wound areas were captured on day 7 post-transplantation, and the necrotic areas were analyzed using the ImageJ software program (NIH)^48^.

### Histological analyses

The skin tissues were collected and fixed overnight with 4% paraformaldehyde following by washing with PBS and soaking in 10% sucrose for 2 h and then 20% sucrose overnight. The wound tissues were embedded in O.C.T compound (Sakura Finetek, Tokyo, Japan) and frozen in liquid nitrogen to make frozen blocks of samples. The frozen wound tissue sections were mounted, stained with hematoxylin and eosin (Wako), and observed under a microscope (Olympus, Kyoto, Japan) to assess the tissue structure.

Immunohistochemical staining was used to analyze the role of AT-MSCs in recruiting inflammatory cells and forming vessels at wound sites. The inflammatory cells recruited to the ischemic area were examined by immunohistochemical staining of the wound tissues with rat anti-mouse CD45 (553078; BD Pharmigen Inc., San Diego, CA, USA), and rat anti-mouse Mac1 (rat anti-mouse CD11b 550282; BD Pharmigen). The neovascularization was analyzed by immunohistochemical staining of wound tissues with rat anti-mouse CD31 (553370; BD Pharmigen), according to the manufacturer’s instructions. The numbers of positive cells were counted in 10 randomly selected fields, and the average was determined.

### Statistical analyses

The data were analyzed by the Mann Whitney U-test using the GraphPad Prism 5 software program (GraphPad Software Inc., San Diego, CA, USA). *P* values of < 0.05 were considered significant. The data were presented as the mean ± standard deviation (SD).

### Data availability

The datasets generated and/or analyzed during the current study are available from the corresponding author on reasonable request.

## Supplementary information

Supplementary Figures.
