# Supplementary material for: Rejuvenation of mesenchymal stem cells by extracellular vesicles inhibits the elevation of reactive oxygen species
Source: Sci Rep. 2020 Oct 14;10:17315. doi: 10.1038/s41598-020-74444-8 (PMC7560871; doi:10.1038/s41598-020-74444-8)
Supplement: Supplementary file 1 — Supplementary Figures. [file 41598_2020_74444_MOESM1_ESM.docx]

**Supplementary information**

**Rejuvenation of Mesenchymal Stem Cells by Extracellular Vesicles Inhibits the**

**Elevation of Reactive Oxygen Species**

Vuong Cat Khanh^1^, Toshiharu Yamashita^1^, Kinuko Ohneda^2^, Chiho Tokunaga^3^, Hideyuki Kato^3^, Motoo Osaka^3^, Yuji Hiramatsu^3^, Osamu Ohneda^1^

*^1^Graduate School of Comprehensive Human Science, Laboratory of Regenerative Medicine and Stem Cell Biology, University of Tsukuba, Tsukuba 305-8575, Japan.*

*^2^Department of Medical Biochemistry, Tohoku University Graduate School of Medicine, Miyagi, Japan.*

^3^*Department of Cardiovascular Surgery, University of Tsukuba.*

Address correspondence to: Osamu Ohneda, MD, PhD, Laboratory of Regenerative Medicine and Stem Cell Biology, University of Tsukuba, 1-1-1 Tsukuba 305-8575, Japan.

E-mail: oohneda@md.tsukuba.ac.jp

**
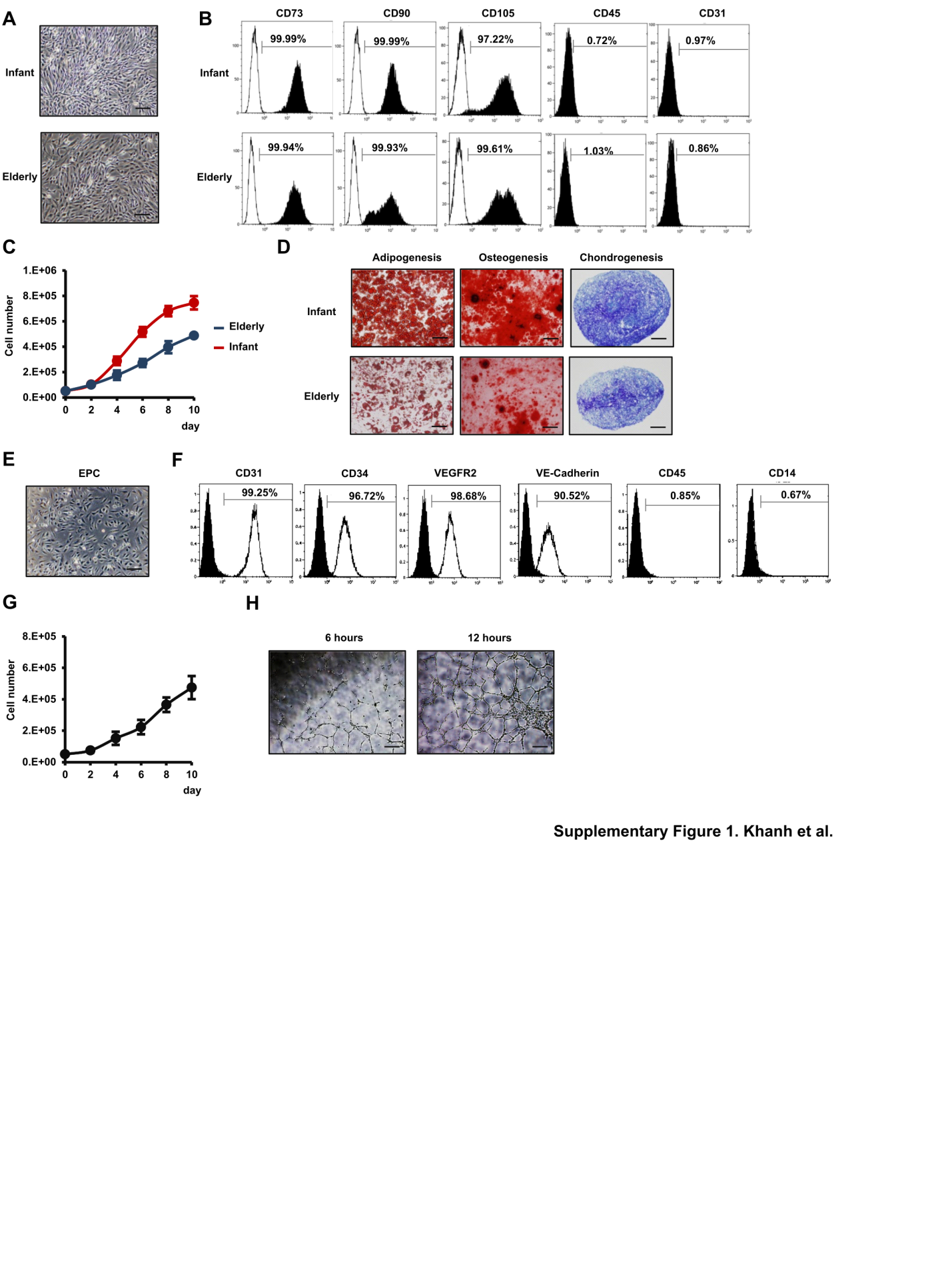
**

**Supplementary Figure 1. Characterization of AT-MSCs and EPCs. A.** Morphology of AT-MSCs, magnification x4, bar indicates 200µm. **B.** MSC marker expression of AT-MSCs by a FACS analysis. **C.** Growth curve of AT-MSCs. **D.** Differentiation of AT-MSCs to adipocytes (magnification x20, bar indicates 50µm), osteocytes (magnification x4, bar indicates 200µm), and chondrocytes (magnification x10, bar indicates 100µm). **E.** Morphology of EPCs, magnification x4, bar indicates 200µm. **F.** EPC marker expression of EPCs by a FACS analysis. **G.** Growth curve of EPCs. **H.** Tube formation of EPCs, magnification x4, bar indicates 200µm. n=5. The experiments were performed in triplicate.


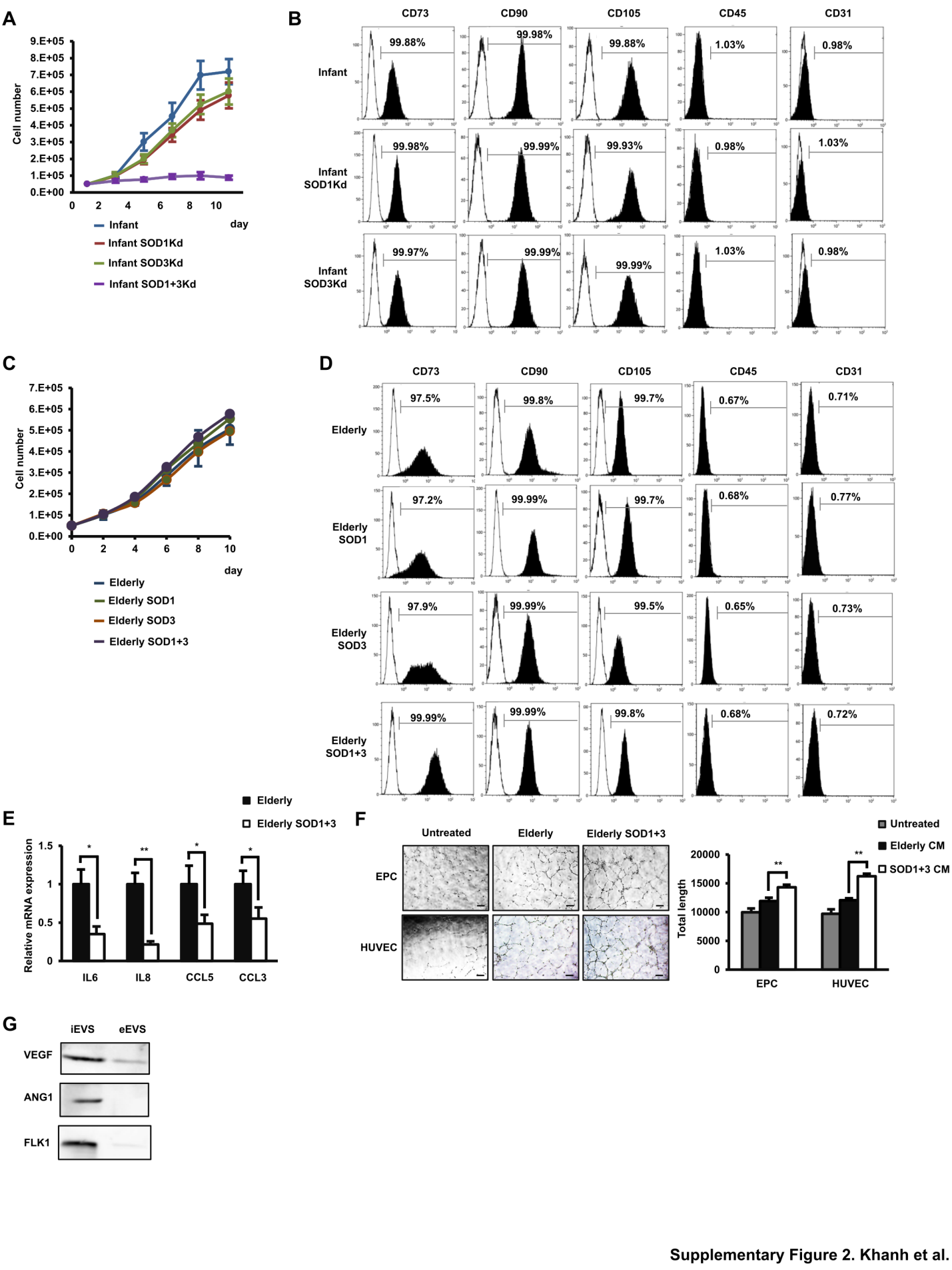


**Supplementary Figure 2. Characterization of AT-MSCs with knockdown or overexpression of SOD1 or/and SOD3. A.** Growth curve of infant AT-MSCs with the knockdown of SOD1, or SOD3, or SOD1 and SOD3. **B.** MSC marker expression of infant AT-MSCs with the knockdown of SOD1 or SOD3. SOD1 Kd: knockdown of SOD1, SOD3 Kd: knockdown of SOD3, SOD1+3Kd: knockdown of SOD1 and SOD3. **C.** Growth curve of elderly AT-MSCs with the overexpression of SOD1, or SOD3, or co-overexpression of SOD1 and SOD3. **D.** MSC marker expression of elderly AT-MSCs with the overexpression of SOD1, or SOD3, or co-overexpression of SOD1 and SOD3. **E.** mRNA expression of pro-inflammatory cytokines in elderly AT-MSCs with the co-overexpression of SOD1 and SOD3. **F.** Tube formation assay of EPCs and ECs under the conditioned medium from elderly AT-MSCs with the co-overexpression of SOD1 and SOD3, magnification x4, bar indicates 200µm. In all above experiments, infant AT-MSCs or elderly AT-MSCs were derived from 3 different donors (n=3). **G.** Protein expression of angiogenic factors in iEVs and eEVs at the same amount of protein (10µg). The data represent the mean ± SD. **P<0.01, *P<0.05. The experiments were performed in triplicate.


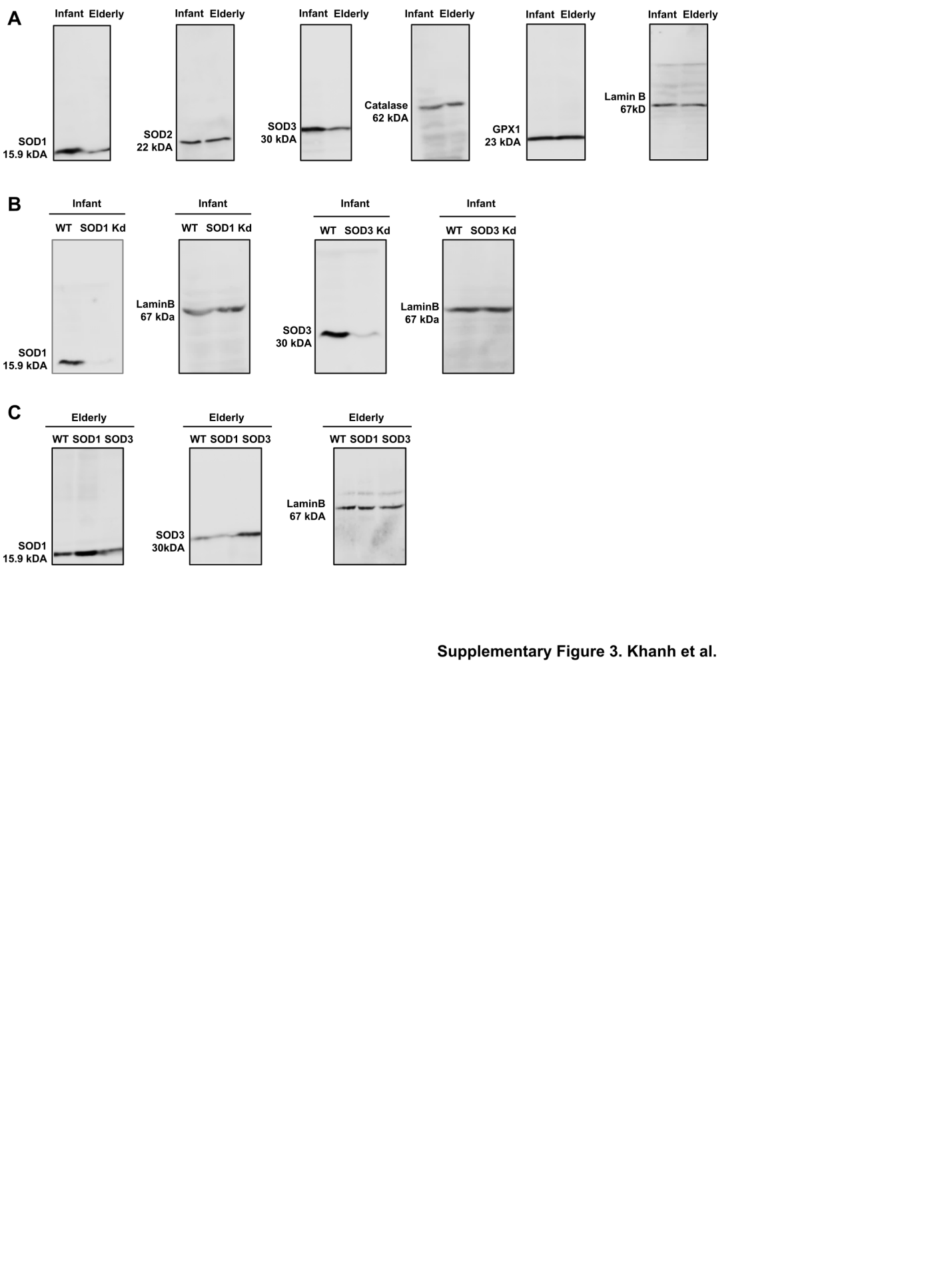


**Supplementary Figure 3. Full-length blots of Western blot figures shown in Figure 2. A.** The protein expression of antioxidant enzymes in infant and elderly AT-MSCs. **B.** The protein expression of wild-type, SOD1 knockdown, and SOD3 knowckdown infant AT-MSCs. **C.**  The protein expression of wild-type, SOD1 overexpressed-, and SOD3 overexpressed-elderly AT-MSCs. The samples were derived from the same experiments that gels and blots were processed in parallel.


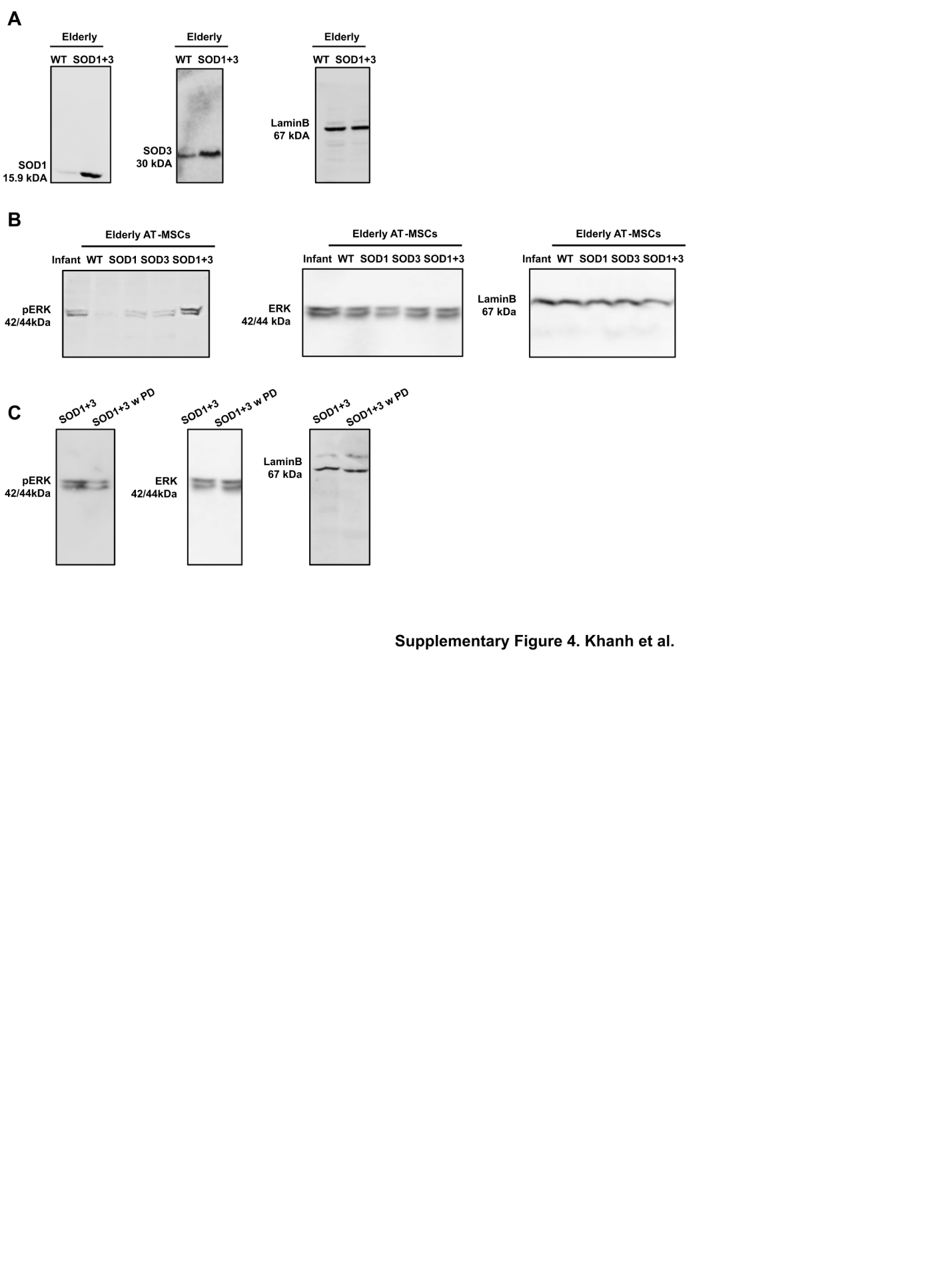


**Supplementary Figure 4. Full-length blots of Western blot figures shown in Figure 3. A.** The protein expression of wild-type elderly AT-MSCs or with the co-overexpression of SOD1 and SOD3. **B.** The protein expression of pERK/ERK in infant AT-MSC, wildtype elderly AT-MSCs, elderly AT-MSCs with the individual overexpression of SOD1 or SOD3 or elderly AT-MSCs with the co-overexpression of SOD1 and SOD3. **C.** The protein expression of pERK/ERK under the presence of a MEK inhibitor.

**
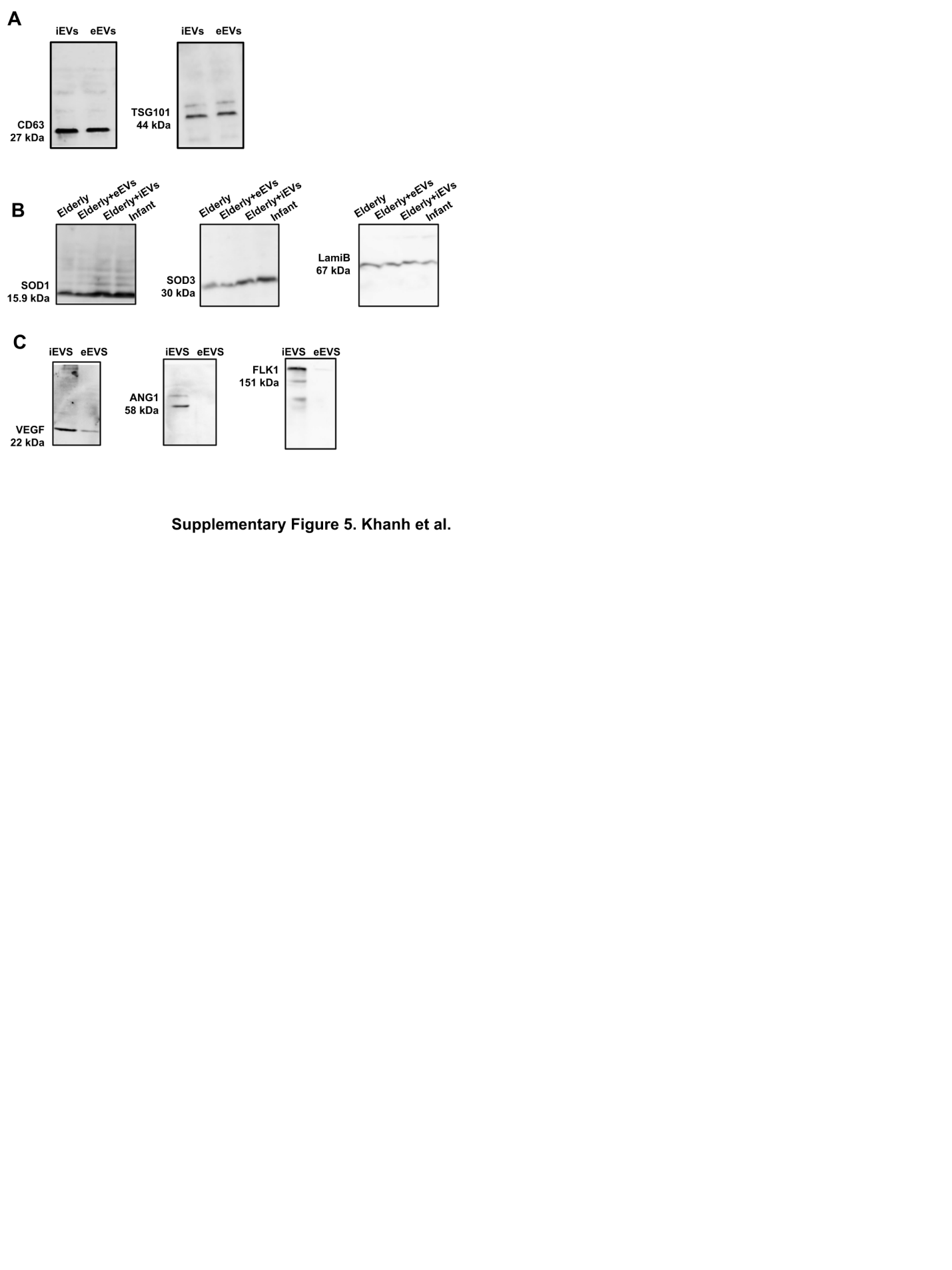
**

**Supplementary Figure 5. Full-length blots of Western blot figures shown in Figure 4 and Supplementary Figure 2G. A.** The marker expression of EVs. **B.** The SOD1 and SOD3 protein expression of elderly AT-MSCs with EV incorporation. **C.** The angiogenic protein exression in iEVs and eEVs at the same amount of protein (10µg). The samples were derived from the same experiments that gels and blots were processed in parallel.

**Supplementary Methods**

**Differentiation of AT-MSCs**

For adipogenesis, AT-MSCs were seed with the number of 5x10^4^ cells in the 4-well plate and cultured in the complete culture medium until reach 90% confluency. Then, AT-MSCs were induced to adipocytes by changing the medium to adipogenic medium containing of IMDM, 10% FBS, 0.1 mM dexamethasone, 2 mg/mL insulin, 500 mM 3-isobutyl-1-methylxanthine (IBMX), and 10 mM indomethacine (Sigma-Aldrich). After 21 days, the formation of adipocytes were confirmed by the Oil red staining. Cells were washed with 1x PBS and fixed with 10% paraformaldehyde, and rinsed with 60% isopropanol. Then, cells were stained with Oil red O (Muto Pure Chemicals, Tokyo, Japan), and the observation of adipocytes was conducted under a microscopy (Olympus) at a magnification x20.

For osteogenesis, AT-MSCs were seed with the number of 5x10^4^ cells in the 4-well plate and cultured in the complete culture medium until reach 90% confluency. Then, AT-MSCs were induced to osteocytes by changing the medium to osteogenic medium containing IMDM, 1% FBS, 0.1 mM dexamethasone, 50 μg/mL human epidermal growth factor (hEGF), 1 M β-glycerol-2-phosphate, and 1 M ascorbic acid (Sigma-Aldrich). After 21 days, cells were washed with 1x PBS, and fixed with 10% paraformaldehyde (Wako) for the Alizarin Red S staining (Kodak, Tokyo, Japan). Then, the formation of osteocytes was observed under a microscopy at a magnification x4.

For chondrogenesis, AT-MSCs were seed with the number of 2.5x10^5^ cells/well in a 96-well plate (MS-9096U, Sumilon, Wako) for 24 hour to form the spheroid. After that, medium was changed to the chondrogenic medium containing IMDM medium supplemented with 1% FBS, 0.1 mM dexamethasone, 100 mM sodium pyruvate (Invitrogen), 1 M ascorbic acid, 50 mg/mL ITS premix (BD Bioscences), 40 μg/mL proline (Sigma-Aldrich), 10 μg/mL TGF-β1 (Wako), and 10 μg/mL BMP-2 (Wako). After 21 days, spheroids were collected, washed with 1x PBS and fixed with 4% paraformaldehyde and embedded in O.C.T compound. Block of O.C.T compound which contained of spheroid samples were sectioned into 7 μm thickness of slides and stained with Toluidine blue (Muto Pure Chemicals).

**Analysis of MSC and EPC marker expression by Fluorescent activated cell sorting (FACs)**

For the analysis of MSC marker expression, AT-MSCs were seeded in a 10-cm dish with a number at 5x10^5^ cells until reach 80% confluency. The cells were harvested, washed with PBS and stained with human antibodies for the analysis of MSC markers, including: phycoerythrin (PE)-labeled anti-CD73 (BD Biosciences), Fluorescein isothiocyanate (FITC)-labeled anti-CD90 (BioLegend), PE-labeled anti-CD105 (BioLegend), allophycocyanine (APC)-labeled anti-CD45 (BD Biosciences), and PE-labeled anti-CD31 (BioLegend). FITC-labeled anti-IgG1 (BD Biosciences), APC-labeled anti-IgG1 (BD Biosciences), and PE-labeled anti-IgG1 (BD Biosciences) were used as the isotype controls. The stained cells were analyzed by a FACS machine (MoFlo XDP; Beckman Coulter).

For the analysis of EPC marker expression analysis, EPCs were seeded in a 10-cm dish with a number at 5x10^5^ cells until reach 80% confluency. The cells were harvested, washed with PBS and stained with human antibodies for the analysis of EPC markers, including: PE-labeled anti-CD31 (BioLegend), FITC-labeled anti-CD34 (BD Biosciences), APC-labeled anti-CD45 (BD Biosciences), PE-labeled anti-CD14 (BioLegend), PE- labeled anti-VEGFR2, and APC- labeled anti-VE-Cadherin (BioLegend). FITC-labeled anti-IgG1 (BD Biosciences), APC-labeled anti-IgG1 (BD Biosciences), and PE-labeled anti-IgG1 (BD Biosciences) were used as the isotype controls. The stained cells were analyzed by a FACS machine (MoFlo XDP; Beckman Coulter).
